# Hippo pathway and NLRP3-driven NETosis in macrophages: Mechanisms of viral pneumoniaaggravation

**DOI:** 10.1038/s41420-025-02556-z

**Published:** 2025-07-14

**Authors:** Bijun Luo, Xiaoxia Wang, Jinyuan Lin, Jianlan Mo, Jia’an Xie, Yanqiong zhou, Jifeng Feng, Linghui Pan

**Affiliations:** 1 Department of Anesthesiology, Guangxi medical university Cancer hospital, Nanning, Guangxi China; 2 Guangxi Clinical Research Center for Anesthesiology, Nanning, Guangxi China; 3 Guangxi Health Commission Key Laboratory of Basic Science and Prevention of Perioperative Organ Disfunction, Nanning, Guangxi China; 4 Guangxi Engineering Research Center for Tissue and Organ Injury and Repair Medicine, Nanning, Guangxi China; 5 Department of Anesthesiology, The Maternal and Child Health Care Hospital of Guangxi Zhuang Autonomous Region, Nanning, Guangxi China

**Keywords:** Viral infection, Transcriptomics

## Abstract

Severe viral infections can precipitate acute lung injury, resulting in significant morbidity and mortality. While NETosis serves as an important defense mechanism against pathogens and viruses, its excessive or dysregulated activation may contribute to pulmonary damage. In this study, elevated levels of NETosis were detected in the peripheral blood of patients with viral pneumonia. To further investigate the relationship between NETosis and virus-induced acute lung injury, a murine model was established using intratracheal administration of poly(I:C), a synthetic analog of double-stranded RNA that mimics viral infection. NETosis biomarkers were assessed in both patients and poly(I:C)-stimulated mice. In addition, we examined the role of the Hippo signaling pathway and its downstream mediators, including inflammatory factors and chemokines. Enhanced NETosis and activation of the Hippo pathway were observed in the lungs of poly(I:C)-treated mice, along with elevated levels of IL-1β in isolated macrophages. These effects were mitigated by Hippo pathway inhibitors. Co-culture experiments confirmed that IL-1β promotes NETosis, while NLRP3, acting downstream of the Hippo pathway, was responsible for IL-1β secretion. Patients with viral pneumonia showed increased NLRP3 and IL-1β expression in monocyte-derived macrophages compared to healthy controls. Overall, our findings indicate that activation of the Hippo pathway in macrophages during poly(I:C) exposure upregulates NLRP3 and IL-1β expression, thereby promoting NETosis and exacerbating virus-induced lung injury. This study highlights a potential therapeutic target to reduce lung damage caused by viral infections.

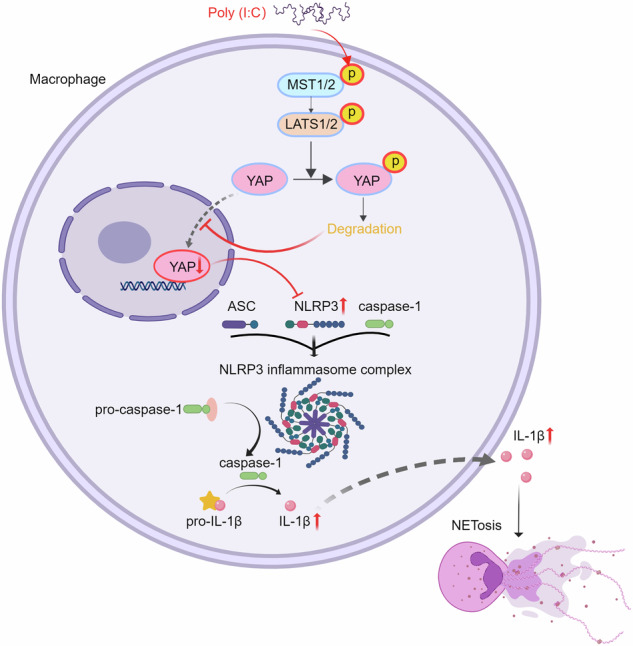

## Introduction

Respiratory viral infections, such as influenza, SARS-CoV, and MERS-CoV, are global public health problems with substantial seasonal and pandemic morbidity and mortality [1–3]. Current treatments primarily focus on supportive care, antiviral medications, and in some cases, the use of corticosteroids. However, these approaches often face limitations regarding efficacy, antiviral resistance, and potential adverse effects [4]. Therefore, it is critical to dive deeper into the underlying mechanisms of virus-host interactions.

The immune microenvironment plays pivotal roles in viral recognition, elimination, and clearance of infected cells during viral lung injury. Innate immune cells such as neutrophils, natural killer (NK) cells, and macrophages rapidly migrated to infection sites where they exert antiviral functions [5]. The immune response remains critical throughout all phases of viral lung damage, from initial antiviral defense to tissue damage repairs [6]. Understanding the dynamic interactions between immune cells within this microenvironment is vital for developing novel therapeutic strategies and improving patient outcomes.

NETosis, the process by which neutrophils release neutrophil extracellular traps (NETs) as part of the innate immune response, plays a critical role in the body’s defense against various pathogens, including viruses [7, 8]. NETs, consisting of DNA fibers adorned with antimicrobial proteins, have been documented to play a pivotal role in trapping and neutralizing virus particles, thus impeding viral replication and dissemination [9]. While NETs can trap and neutralize viruses, excessive or dysregulated NET formation can lead to increased pulmonary inflammation and tissue damage [10, 11]. However, the molecular mechanisms regulating NETosis in viral pneumonia and their contribution to disease severity remain poorly understood.

A critical gap in our knowledge is how cellular signaling pathways coordinates the inflammatory responses that trigger NET formation during viral lung injury. The hippo signaling pathway, primarily known for its role in organ size control and tissue homeostasis, has recently been implicated in immune regulation [12]. However, its potential role in NETosis and viral pneumonia pathogenesis has yet been studied.

In the present study, we hypothesized that the Hippo pathway might regulate inflammatory responses in macrophages during viral pneumonia, thereby influencing NETosis and disease severity. Using a poly(I:C)-induced viral lung injury mouse model and clinical samples from viral pneumonia patients, we identified a previously unrecognized mechanism whereby the Hippo pathway is activated in macrophages and drives NLRP3 expression, which leads to IL-1β production and subsequent NETosis, thereby exacerbating the severity of pneumonia.

Our findings reveal a novel Hippo-NLRP3-IL-1β-NETs axis that contributes to viral pneumonia pathogenesis. This provides new insights into the cellular and molecular mechanisms underlying viral lung injury. By identifying the Hippo pathway and NLRP3 as potential therapeutic targets for modulating excessive inflammation and NETosis, our work offers promising directions for developing more effective treatments for viral pneumonia.

## Results

### NETosis is associated with inflammation in patients with viral pneumonia

**Fig. 1:**
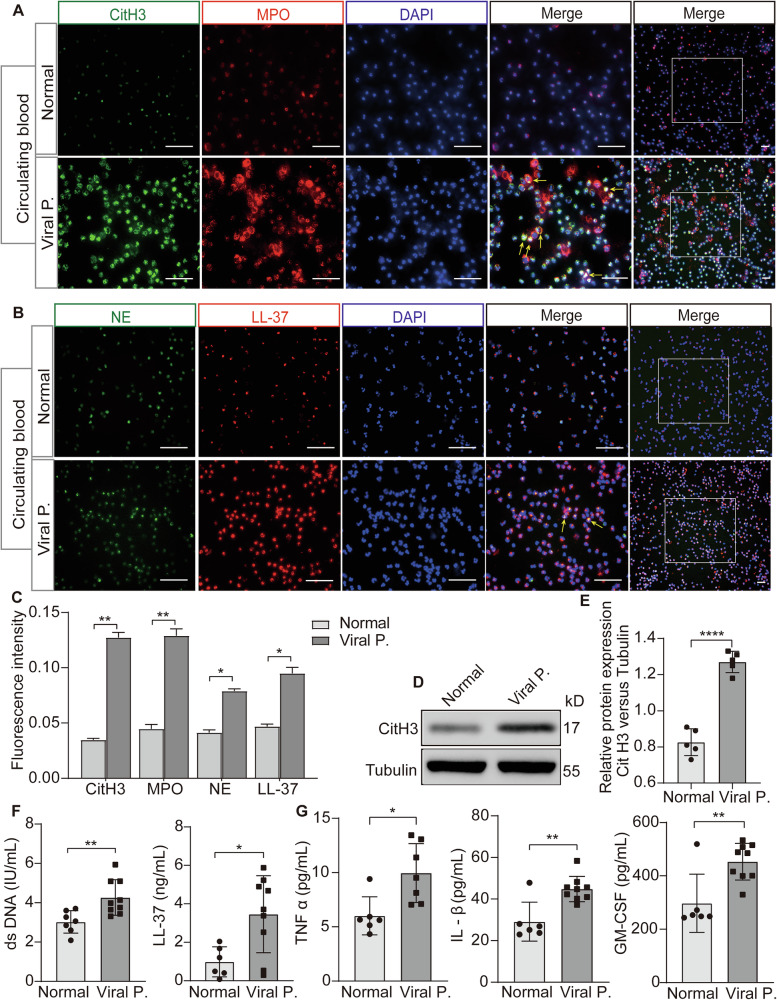
NETosis is involved in viral pneumonia with excessive inflammatory cytokines. **A**, **B** Comparative analysis of NETosis biomarkers in neutrophils from peripheral blood of patients with viral pneumonia versus healthy volunteers via immunofluorescence. **C** Graphic presentations of fluorescence mean densities of NETosis biomarkers. **D** Assessment of CitH3 protein expression in whole blood sample from patients with viral pneumonia and healthy volunteers using western blotting. **E** Quantitative analysis of the protein CitH3 relative to Tubulin. **F** Quantification of serum dsDNA and LL-37 levels conducted through Enzyme linked immunosorbent assay (ELISA). **G** Evaluation of serum pro-inflammatory cytokines, including TNF-α, IL-1β, and GM-CSF, via ELISA. All data are representative as means ± s.e.m of three independent experiments. Student’s *t* test for A-G; *p < 0.05; **p < 0.01; ***p < 0.001. Scale bar = 50 μm.

To evaluate the role of NETosis in viral pneumonia, NETosis biomarkers were measured, incorporating citrullinated histone H3 (CitH3), myeloperoxidase (MPO), neutrophil elastase (NE), and LL-37 in the neutrophil from peripheral blood, as well as dsDNA and LL-37 in the serum of individuals suffering from viral pneumonia and healthy participants [13–15]. The viral pneumonia patients showed increased levels of CitH3, MPO, NE, and LL37 compared to healthy individuals (Fig. 1A–C). The levels of the CitH3 protein in patients suffering from viral pneumonia were also elevated when compared to those found in healthy individuals (Fig. 1D, E), and serum dsDNA and LL-37 levels were both increased in these patients (Fig. 1F). Additionally, serum inflammatory cytokine levels, such as IL-1β, TNF-α, and GM-CSF, were higher in these patients than in healthy volunteers (Fig. 1G), suggesting that significant inflammation was observed in patients with viral pneumonia. Taken together, NETosis may be associated with a high expression of pro-inflammatory factors, which are potential risk factors for viral pneumonia.

### NETosis is activated in mouse acute lung injury following poly(I:C) stimulation

**Fig. 2:**
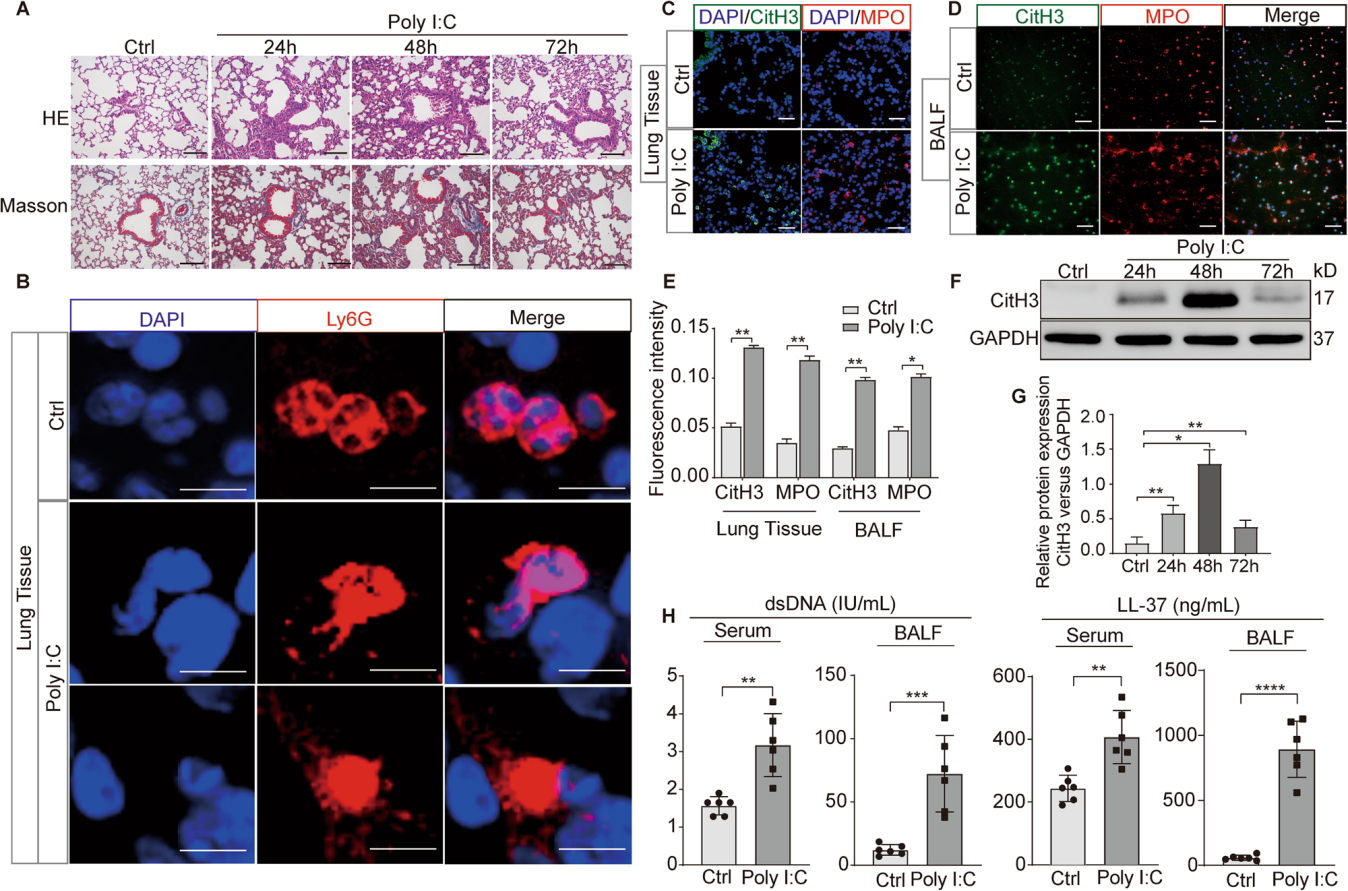
Role of NETosis in mouse acute lung injury induced by poly(I:C) stimulation. **A** H&E and Masson staining of lung tissues from mice subjected to poly(I:C) stimulation versus control vehicle, respectively. Scale bar = 50 μm. **B**–**D** Comparative analysis of NETosis biomarkers in lung tissues from mice with or without poly(I:C) stimulation via immunofluorescence. B, Scale bar = 5 μm; C-D, Scale bar = 50 μm. **E** Graphic presentations of fluorescence mean densities of CitH3 and MPO. **F** Assessment of CitH3 protein in lung tissues from mice with or without poly(I:C) stimulation using western blotting. **G** Quantitative analysis of the protein CitH3 relative to GAPDH. **H** Serum concentrations of dsDNA and LL-37 were assessed by ELISA. All data are representative as means ± s.e.m of three independent experiments. Student’s *t* test for (**A**–**F**); *p < 0.05; **p < 0.01; ***p < 0.001.

To investigate the relationship between NETosis and viral pneumonia, C57BL/6 mice underwent intranasal exposed to poly(I:C), resulting in evident pathological injury and the accumulation of collagen fibers (Fig. 2A). Poly(I:C) was selected because it mimics the double-stranded RNA produced during viral replication of many respiratory viruses, provides a consistent and reproducible model of viral-like lung injury, and allows for the study of innate immune responses without the biosafety concerns associated with live viral pathogens. Neutrophils exhibited membrane rupture, releasing network structures containing DNA and antimicrobial proteins in mice with viral pneumonia (Fig. 2B). Relative to the control mice, both CitH3 and MPO were exhibited at elevated levels in the pulmonary tissues of mice afflicted with poly(I:C) induced lung injury (Fig. 2C–E). Compared with the control group, the relative expression of CitH3 protein in mice with viral pneumonia at 24 h, 48 h, and 72 h after poly(I:C) exposure was upregulated, peaking at 48 h post-exposure (Fig. 2F, G). Additionally, dsDNA and LL-37 levels were higher in the serum and BALF of mice suffering from poly(I:C)-induced lung injury compared to those in the control group (Fig. 2H). This shows that NETosis is significantly activated in mice with poly(I:C) induced lung injury, as indicated by lung injury and heightened levels of NETosis markers, peaking at 48 h post-exposure.

### Hippo pathway is activated in macrophages from poly(I:C) stimulated mice

**Fig. 3:**
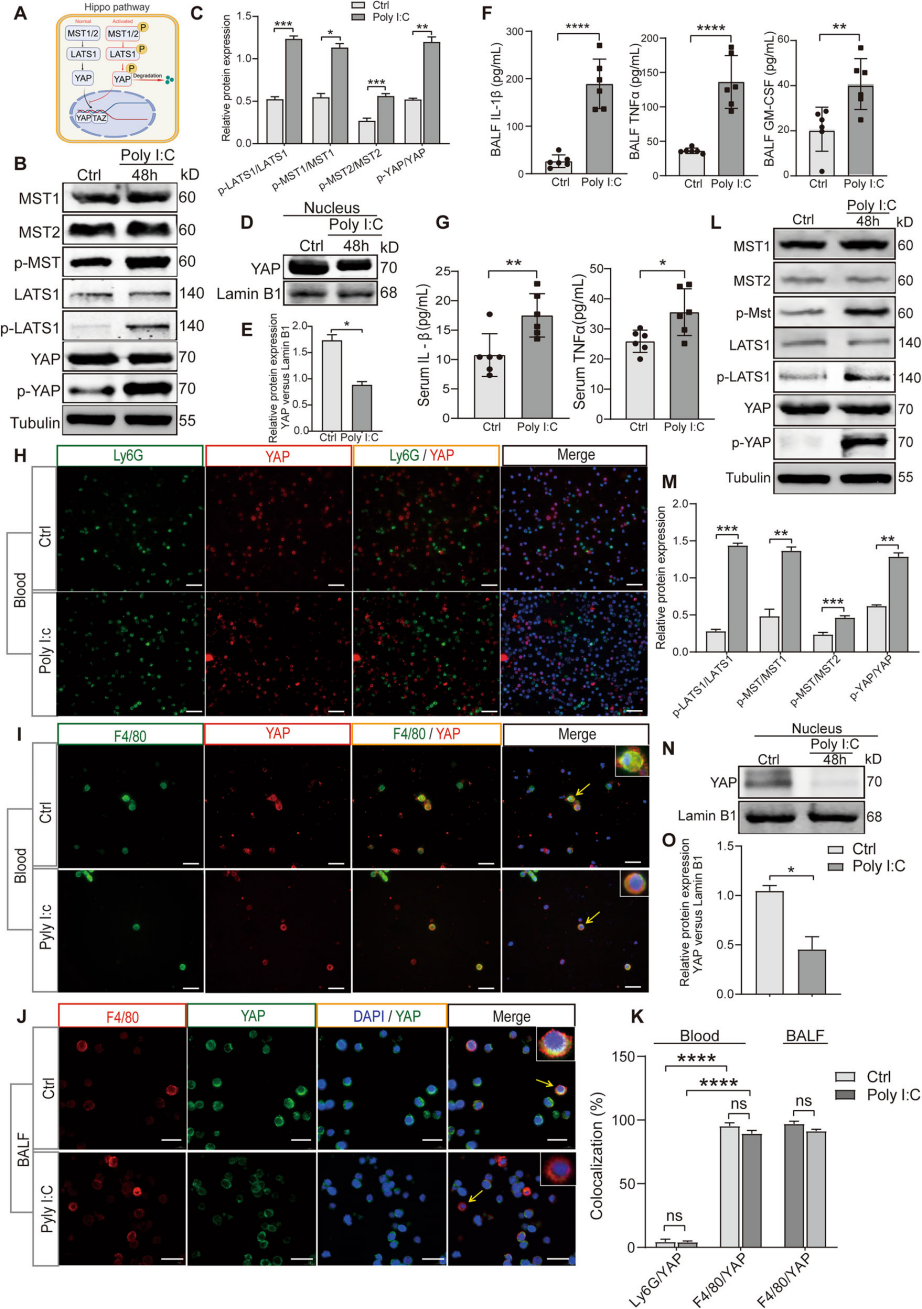
Activation of the Hippo pathway in macrophages following poly(I:C) stimulation in mice. **A** A simplified schematic diagram illustrating the interactions between components of the Hippo pathway. **B** Expression of Hippo pathway expression in lung tissues from mice treated with poly(I:C) compared to untreated controls. **C** The protein expression of Hippo pathway were quantified using densitometry on ImageJ. Phospho-LATS1 and LATS1, phospho-MST and MST1, phospho-MST and MST2, phospho-YAP and YAP protein were normalized to Tubulin and the ratio is presented. **D** Relative expression of YAP protein in lung tissues from mice treated with or without poly(I:C) was assessed after nuclear-plasma separation. **E** Quantitative analysis of the protein YAP in nuclear relative to Lamin B1. **F** The levels of inflammatory cytokines and chemokines in BALF. **G** The levels of inflammatory cytokines and chemokines in serum. **H** YAP and neutrophils colocalization in peripheral blood of mice infected with poly(I:C). Scale bar = 50 μm. **I**, **J** Colocalization analysis of YAP with macrophages in peripheral blood (**H**) and BALF (**I**) from poly(I:C) stimulated mice. Scale bar = 20 μm. **K** Quantification of the colocalization between Ly6G or F4/80 and YAP. **L** Expression of the Hippo pathway in macrophages from mice treated with or without poly(I:C). **M** Quantification of phospho-LATS1 and LATS1, phospho-MST and MST1, phospho-MST and MST2, phospho-YAP and YAP protein in macrophages from mice were normalized to Tubulin and the ratio is presented. **N** Relative expression of YAP protein in macrophages following nuclear-plasma separation from mice treated with or without poly(I:C). **O** Quantitative analysis of the protein YAP in nuclear relative to Lamin B1. All data are representative as means ± s.e.m of three independent experiments. Student’s *t* test for (**A**–**I**); *p < 0.05; **p < 0.01; ***p < 0.001; ****p < 0.0001.

Although the Hippo pathway is primarily associated with cell fate decisions, proliferation, migration, death, and organ size control [16], the Hippo pathway is critical in pulmonary inflammatory diseases [17], and its specific role in virus-induced lung injury remains unclear. Within the pulmonary tissue of mice stimulated with poly(I:C), the Hippo pathway is activated (Fig. 3A–E), leading to increased levels of inflammatory factors and chemokines (Fig. 3F, G). Yes-associated protein (YAP), a pivotal molecule within the Hippo pathway, undergoes phosphorylation and cytoplasmic retention upon pathway activation, resulting in diminished nuclear YAP levels. Immunofluorescence was used to examine the co-localization of YAP with neutrophils (Fig. 3H) or macrophages (Fig. 3I, J), revealing that YAP co-localized with macrophages rather than neutrophils (Fig. 3K). The YAP and Hippo pathways were assessed through the nucleocytoplasmic separation of macrophages extracted from the bronchoalveolar lavage fluid (BALF) of mice stimulated with poly(I:C). The findings verify that the stimulation of the Hippo pathway triggered by poly(I:C) stimulation takes place chiefly within macrophages (Fig. 3L–O).

### Hippo pathway in macrophages is activated to promote NETosis in murine viral pneumonia following poly(I:C) stimulation

**Fig. 4:**
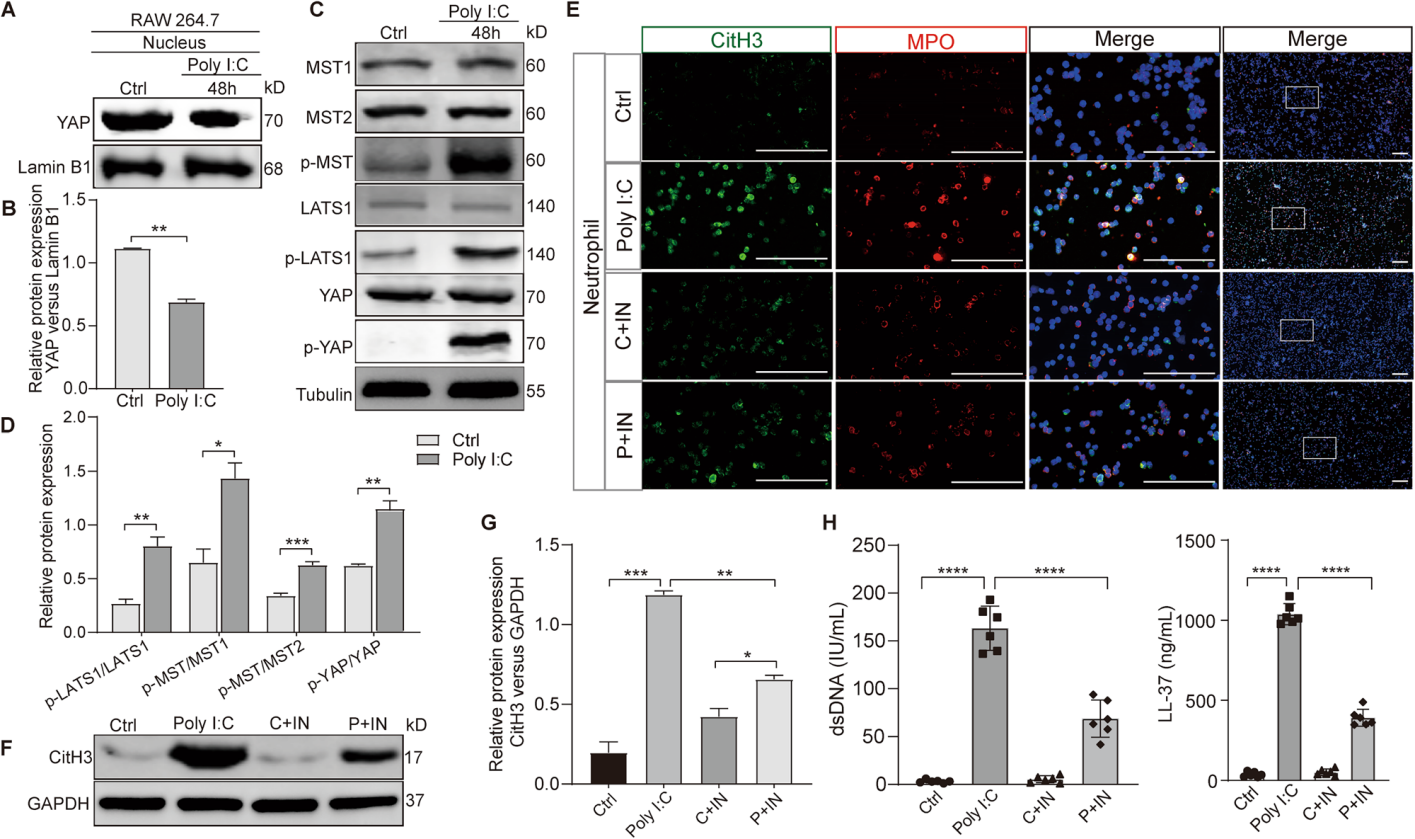
Activation of the Hippo Pathway in Macrophages Enhances NETosis under poly(I:C) stimulation. **A** Relative expression of nuclear YAP protein in RAW246.7 cells treated with or without poly(I:C) was assessed, following nuclear-plasma separation. **B** Quantitative analysis of the protein YAP in nuclear relative to Lamin B1. **C** Hippo pathway expression in RAW246.7 cells treated with or without poly(I:C). **D** Quantification of phospho-LATS1 and LATS1, phospho-MST and MST1, phospho-MST and MST2, phospho-YAP and YAP protein in macrophages from mice were normalized to Tubulin and the ratio is presented. **E**–**H** Neutrophils were cultured with conditioned medium collected from RAW 264.7 cells that had been treated under different conditions. Neutrophils were collected and level of CitH3 and MPO were assessed using immunofluorescence (**E**) CitH3 protein expression was assessed using Western blotting (**F**, **G**). dsDNA and LL-37 levels were evaluated by ELISA (**H**). Note: IN, Lats-IN-1 is a potent and ATP-competitive inhibitor of LATS1 and LATS2 kinases. All data are representative as means ± s.e.m of three independent experiments. Student’s *t* test for (**A**–**H**); *p < 0.05; **p < 0.01; ***p < 0.001; ****p < 0.0001. Scale bar = 20 μm.

Investigating how activation of the Hippo pathway in macrophages following poly(I:C) stimulation promotes NETosis in murine viral pneumonia. In RAW264.7 cells stimulated with poly(I:C), the expression of nuclear YAP was decreased, while the Hippo pathway was activated (Fig. 4A–D). In the co-culture of conditioned medium from RAW246.7 cells and neutrophils, NETosis biomarkers in co-cultured neutrophils were upregulated under medium from poly(I:C) stimulated RAW246.7 cells. These biomarkers were abolished under medium from poly(I:C) stimulated RAW246.7 cells after the administration of Hippo pathway inhibitors (Fig. 4E–H). This suggests that Hippo pathway activation in macrophages stimulated with poly(I:C) promotes NETosis.

### In vitro experiments confirm that IL-1β plays a crucial role in facilitating the interaction between macrophages and NETs

**Fig. 5:**
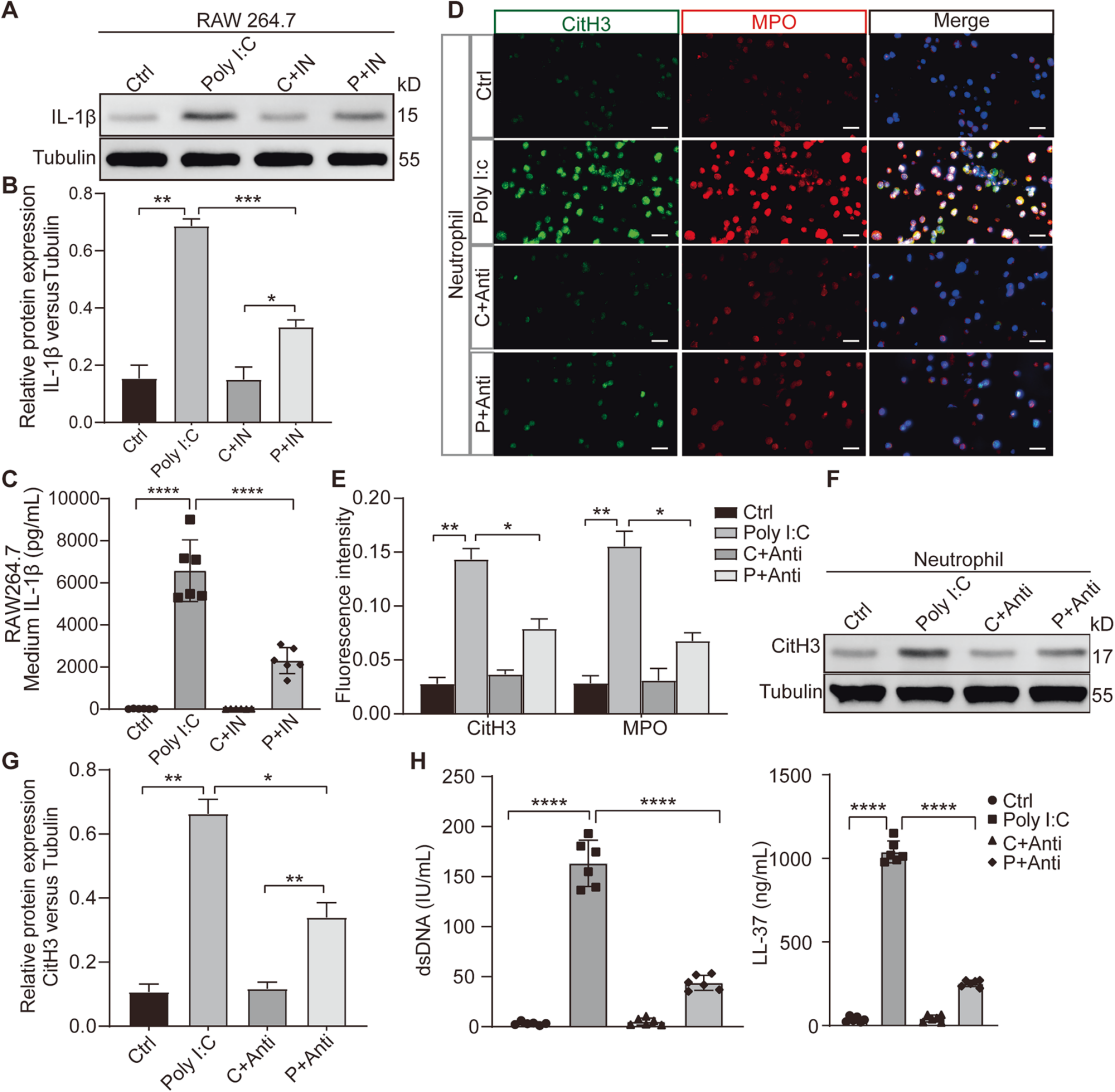
IL-1β mediates the macrophages inducing NETosis under poly(I:C) stimulation. **A** Relative expression of IL-1β protein in RAW246.7 cells treated with poly(I:C) and Hippo pathway inhibitors was assessed. **B** Quantitative analysis of the protein IL-1β relative to Tubulin under different conditions. **C** Measurement of IL-1β level in culture supernatants from RAW246.7 cells treated with poly(I:C) and Hippo pathway inhibitors Lats-IN-1 by ELISA. **D** NETosis biomarker levels in the cocultures of neutrophils and conditioned medium for RAW246.7 cells under poly(I:C) stimulation and neutralized IL-1β antibody were assessed by immunofluorescence. **E** Graphic presentations of fluorescence mean densities of CitH3 and MPO under different conditions. **F** Evaluation of CitH3 protein expression in the co-cultures of neutrophils and conditioned medium for RAW246.7 cells under poly(I:C) stimulation and neutralized IL-1β antibody by western blotting. **G** Quantitative analysis of the protein CitH3 relative to Tubulin. **H** dsDNA and LL-37 levels in the cocultures of neutrophils and conditioned medium for RAW246.7 cells under poly(I:C) stimulation and neutralized IL-1β antibody were evaluated by ELISA. Note: IN, Lats-IN-1 is a potent and ATP-competitive inhibitor of LATS1 and LATS2 kinases. All data are representative as means ± s.e.m of three independent experiments. Student’s *t* test for (**A**–**H**); *p < 0.05; **p < 0.01; ***p < 0.001; ****p < 0.0001. Scale bar = 20 μm.

Several cytokines have been found to stimulate or promote NETosis formation. For example, the activation of the Hippo pathway induces the expression of IL-1β, a critical pro-inflammatory factor implicated in the promotion of NETosis [18]. IL-1β levels were increased in RAW246.7 cells that received poly(I:C) treatment, a change abolished in RAW246.7 cells treated with poly(I:C) and Hippo pathway inhibitors (Fig. 5A–C). In the co-culture of neutrophils and conditioned medium from RAW246.7 cells under poly(I:C) stimulation and the neutralized IL-1β antibody, the promotion of NETosis by IL-1β was attenuated, suggesting that IL-1β is the key molecule mediating macrophage-induced NETosis (Fig. 5D–H).

### Validation of IL-1β as the critical mediator facilitating the interaction between macrophages and NETosis in vivo

**Fig. 6:**
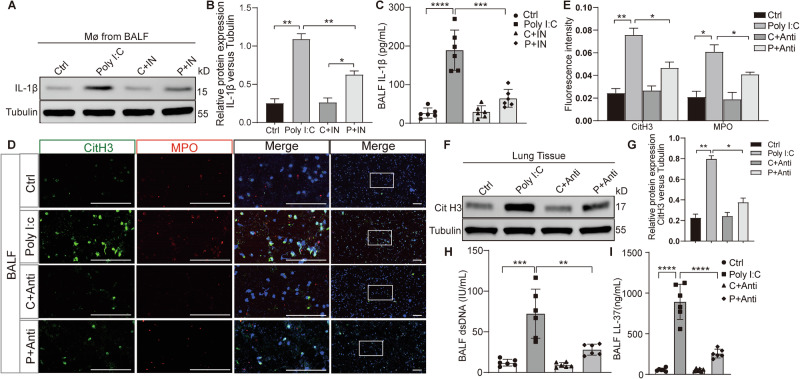
IL-1β mediates the macrophages inducing NETosis under poly(I:C) stimulation in vivo. **A** Evaluation of IL-1β protein expression in macrophages isolated from the BALF of mice with poly(I:C) infection and treated with Hippo pathway inhibitors. **B** Quantitative analysis of the protein IL-1β relative to Tubulin under different conditions. **C** Measurement of Serum IL-1β level from mice with poly(I:C) infection and treated with Lats-IN-1 by ELISA. **D** NETosis biomarker levels from cells in the BALF of mice with poly(I:C) stimulation and neutralized IL-1β antibody were assessed by immunofluorescence. **E** Graphic presentations of fluorescence mean densities of CitH3 and MPO under different conditions. **F** CitH3 protein expression in the lung tissues from mice with poly(I:C) stimulation and neutralized IL-1β antibody was evaluated by western blotting. **G** Quantitative analysis of the protein CitH3 relative to Tubulin. **H**, **I** dsDNA and LL-37 levels in the BALF from mice infected with poly(I:C) and treated with neutralized IL-1β antibody, evaluated by ELISA. Note: IN, Lats-IN-1 is a potent and ATP-competitive inhibitor of LATS1 and LATS2 kinases. All data are representative as means ± s.e.m of three independent experiments. Student’s *t* test for (**A**–**I**); *p < 0.05; **p < 0.01; ***p < 0.001; ****p < 0.0001. Scale bar = 20 μm.

In vivo experiments revealed that IL-1β levels were increased in isolated macrophages in the BALF of mice stimulated with poly(I:C), however, administration of a Hippo pathway inhibitor simultaneously reduced IL-1β levels (Fig. 6A–C). Mice treated with poly(I:C) and neutralizing IL-1β antibody showed an inhibition of NETosis (Fig. 6D–H). These confirmed IL-1β as a key mediator linking macrophages to NETosis, where its elevation following poly(I:C) stimulation and reduction by Hippo pathway inhibition correlates with NETosis modulation.

### Transcriptome sequencing identified NLRP3 downstream of the Hippo pathway that mediates IL-1β secretion and NETosis

**Fig. 7:**
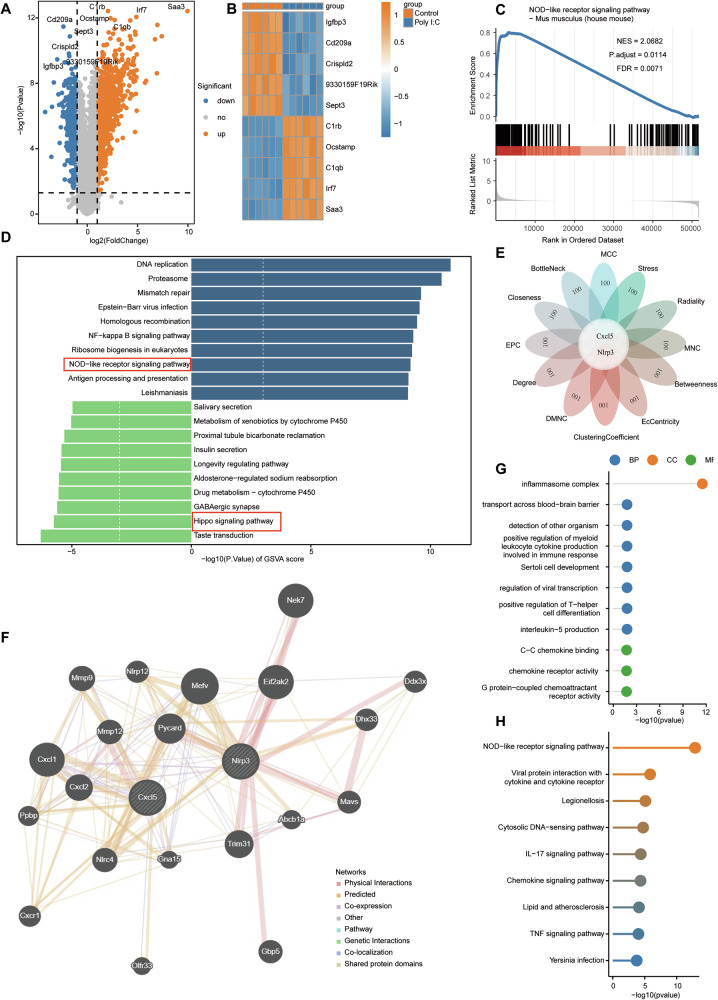
Transcriptome analysis of lung tissues from mice treated with poly(I:C). **A** Volcanic map describes DEGs between the samples of the virus-induced lung injury and control group. Red, blue, and gray dots represent gene expression levels associated with upregulation, downregulation, and no significant expression, respectively. **B** Heat map showed the top 5 up-regulated and down-regulated DEGs. **C** GSEA analysis showed that NOD like receptor signaling pathway was significantly enriched in the virus-induced lung injury. **D** The top 20 pathways with significant differences in GSVA. **E** Hub genes obtained from the PPI network, including Ccxl5 and Nlrp3. **F** Co-expression network diagram of hub genes. **G**, **H** GO and KEGG analysis of co-expressed hub genes.

To delve deeper into the process through which the Hippo pathway facilitates the secretion and discharge of IL-1β, lung tissues from poly(I:C) stimulated mice and PBS as controls were harvested for transcriptome sequencing. Compared to the control group, there were 1625 differentially expressed genes (DEGs), including 1027 upregulated DEGs and 598 downregulated DEGs in poly(I:C) stimulated mice (*p* < 0.05) (Fig. 7A). The top five upregulated DEGs were C1rb, Ocstamp, C1qb, Irf7, and Saa3, whereas the top five downregulated DEGs included Igfbp3, Cd209a, Crispld2, 9330159F19Rik, and Sept3 (Fig. 7B). These DEGs were enriched in the NOD receptor signaling pathway (NES = 2.0682, P.adjust = 0.0114, FDR = 0.0071) using GSEA (Fig. 7C). Analysis of KEGG pathways also indicated that these DEGs were enriched in DNA replication, proteasomes, the Hippo signaling pathway, and taste transduction (Fig. 7D). Sequencing data demonstrate the activation of the Hippo pathway in poly(I:C) stimulated lung tissue, further substantiating prior findings that the Hippo pathway indeed plays a role in poly(I:C) induced lung injury. To pinpoint central genes within the PPI network, the intersection of the top 100 genes in all 12 CytoHubba algorithms was used to obtain two hub genes, Cxcl5 and NLRP3 (Fig. 7E). NLRP3 plays a significant role in the maturation and release of IL-1β, suggesting that the Hippo pathway may facilitate IL-1β formation and release through the upregulation of NLRP3 expression [19, 20]. Another PPI network for hub genes was constructed using the GeneMANIA database (Fig. 7F). GO and KEGG analyses were performed on 22 genes (including two hub genes, 20 genes related to hub genes, and 232 connections) (Fig. 7G–H). These core genes were also found to be abundant in the NOD receptor signaling pathway.

### Hippo pathway regulates IL-1β secretion in macrophages via NLRP3

**Fig. 8:**
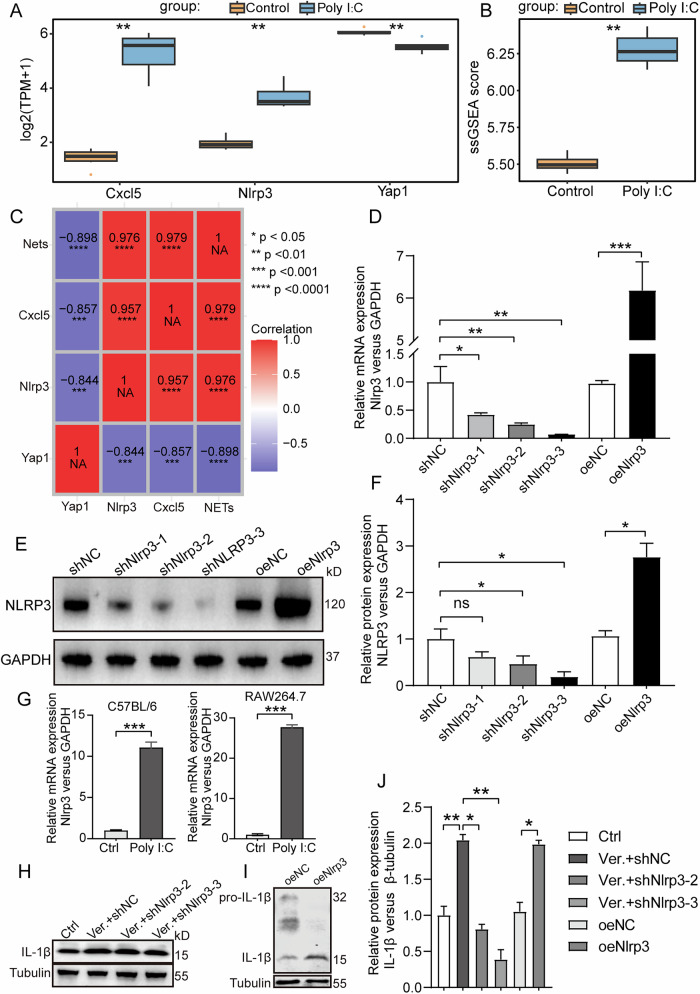
Hippo pathway regulates macrophage IL-1β secretion via NLRP3. **A** Box plot of the expression levels of Cxcl5, Nlrp3, and Yap1 between the virus-induced lung injury and control group. **B** Box graph of NETosis related genes using ssGSEA enrichment scores between the virus-induced lung injury and control group. **C** Correlation heat map. **D**–**F** The efficiency of knockdown and overexpression of NLRP3 via lentivirus vectors were assessedusing PCR and Western blotting detection. **G** Levels of NLRP3 mRNA in macrophages isolated from the BALF of mice and in RAW264.7 cells with or without poly(I:C) stimulated. **H**, **I** L-1β expression in macrophages with or without NLRP3 regulation was evaluated using Western blotting. **J** Quantitative analysis of the protein IL-1β relative to Tubulin under different conditions. All data are representative as means ± s.e.m of three independent experiments. Student’s *t* test for (**A**–**I**); *p < 0.05; **p < 0.01; ***p < 0.001; ****p < 0.0001. Scale bar = 20 μm.

In the lung tissues of mice with poly(I:C) induced lung injury, there was a higher level of Cxcl5 and NLRP3 expression relative to the control group, whereas the expression of YAP1 was reduced. (Fig. 8A). The levels of NETs in the lung tissues of mice with poly(I:C) induced lung injury were elevated compared to the control group, in contrast to the levels of neutrophil immune infiltration (Fig. 8B). Additionally, the expression of Cxcl5 and NLRP3 showed a positive correlation with scores of NET, while the expression of YAP1 was found to have a negative relationship with NET scores (Fig. 8C). This suggests that the Hippo pathway may be activated via the upregulation of Cxcl5 and NLRP3 to promote NETosis. NLRP3, which stands for NOD-like receptor family with a pyridine domain containing 3, plays a crucial role in inflammasomes by triggering the transformation of precursors to pro-inflammatory cytokines, such as IL-1β (pro-IL-1β), to their mature active forms. Therefore, the activation of the Hippo pathway in macrophages may be associated with IL-1β gene release via NLRP3 gene upregulation. Lentivirus vectors, with knocked down and overexpressed NLRP3, were successfully constructed (Fig. 8D–F). The mRNA expression of NLRP3 were regulated in macrophages isolated from the BALF of mice and in RAW264.7 cells with or without poly(I:C) stimulated (Fig. 8G). The activation of the Hippo pathway modulates NLRP3 expression and IL-1β secretion in macrophages in vitro (Fig. 8H–J).

### Expression differences exist in the Hippo/NLRP3/IL-1β pathway in clinical samples

**Fig. 9:**
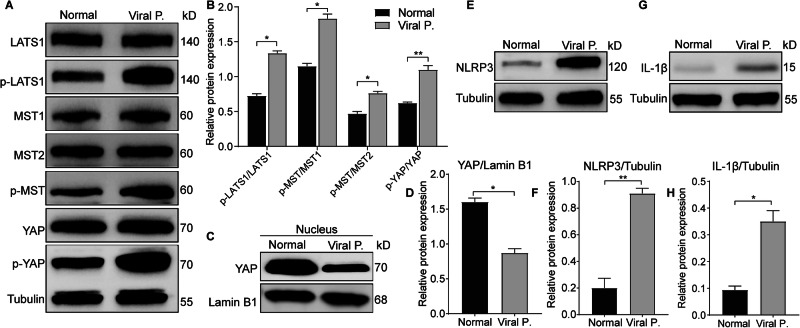
Hippo/NLRP3/IL-1β pathway is activated in patients with viral pneumonia. **A** Hippo pathway expression in mononuclear macrophages isolated from peripheral blood of patients with viral pneumonia and healthy individuals. **B** Relative expression of YAP protein in mononuclear macrophages isolated from peripheral blood of patients with viral pneumonia and healthy individuals was assessed after nuclear-plasma separation. **C** NLRP3 expression in mononuclear macrophages isolated from peripheral blood of patients with viral pneumonia and healthy individuals. **D** Evaluation of IL-1β levels in mononuclear macrophages isolated from peripheral blood of patients with viral pneumonia and healthy individuals. All data are representative as means ± s.e.m of three independent experiments. Student’s *t* test for (**A**–**I**); *p < 0.05; **p < 0.01; ***p < 0.001; ****p < 0.0001. Scale bar = 20 μm.

To examine the function of the Hippo/NLRP3/IL-1β pathway, monocyte macrophages were procured from the circulating blood of individuals suffering from viral pneumonia and healthy volunteers. The Hippo pathway was activated in monocyte-macrophages from patients with viral pneumonia (Fig. 9A–D), and both NLRP3 and IL-1β proteins were highly expressed in these patients compared to those in healthy volunteers (Fig. 9E–H).

## Discussion

The research topic revolves around viral pneumonia, a severe respiratory condition caused primarily by viral infections, which has garnered significant attention due to its high morbidity and mortality rates. Viral pneumonia can lead to acute lung injury (ALI), characterized by widespread inflammation in the lungs, resulting in impaired gas exchange and potential organ failure [21, 22]. The immune response to viral infections plays a critical role in disease progression, with the activation of various immune cells, including macrophages and neutrophils, contributing to both protective and pathogenic effects [21, 22]. Understanding the interplay between viral pathogens and host immune responses is essential for developing effective therapeutic strategies to mitigate the impact of viral pneumonia and improve patient outcomes.

Previous studies have established that YAP promotes NLRP3 inflammasome activation and modulates inflammatory responses, indicating that pharmacological manipulation of the Hippo pathway may offer novel avenues for treatment in conditions characterized by dysregulated inflammation [23, 24]. This study investigates the mechanisms by which the Hippo signaling pathway interacts with NLRP3-driven NETosis in macrophages, particularly in the context of exacerbated viral pneumonia. Our results demonstrate that the inhibition of the Hippo pathway leads to heightened activation of the NLRP3 inflammasome, as evidenced by increased expression levels of NLRP3 and IL-1β in macrophages. This indicates that the Hippo signaling pathway serves as a vital regulatory mechanism that maintains the balance of immune responses during viral infections. The dysregulation of this pathway not only enhances the inflammatory response but also promotes NETosis, resulting in exacerbated lung injury and disease progression in viral pneumonia. These observations align with existing literature that highlights the significance of the Hippo pathway in immune regulation and its potential as a therapeutic target for inflammatory diseases [18, 20].

Furthermore, the intricate relationship between the Hippo pathway and various signaling cascades, particularly those involving NLRP3, underscores the complexity of immune regulation in viral pneumonia. By elucidating the specific mechanisms through which Hippo signaling modulates NLRP3 activation, we provide insights into potential therapeutic interventions aimed at mitigating excessive inflammation and tissue damage. The interplay between YAP/TAZ and NLRP3 may represent a pivotal axis in this context, suggesting that targeting this interaction could enhance therapeutic strategies against viral pneumonia. Furthermore, interactions between Hippo signaling and adaptive immune cells, such as T cells and dendritic cells, could play a crucial role in shaping the overall immune landscape in viral pneumonia [25, 26]. Future studies should explore whether these immune pathways interact with Hippo signaling to modulate disease severity and progression.

In addition to the NLRP3 inflammasome, other inflammasomes may also be involved in the regulation of immune responses during viral pneumonia. The crosstalk between these inflammasomes and Hippo signaling remains an area of interest, as NLRC4 has been implicated in pathogen recognition and the amplification of inflammatory responses [27].

Another important consideration is whether the Hippo-NLRP3-NETosis axis is a universal mechanism across different types of viral infections. While our study focuses on viral pneumonia, it is possible that similar interactions occur in other viral infections affecting different organ systems. For example, in viral myocarditis and viral hepatitis, dysregulated inflammation is a key driver of tissue damage. Investigating whether Hippo signaling influences inflammasome activation and NETosis in these diseases could provide broader insights into the role of this pathway in viral immunopathology. Understanding the commonalities and differences across viral infections will be essential for designing targeted therapeutic strategies that are effective across multiple diseases.

Despite this therapeutic potential, significant challenges remain. Systemic inhibition could impair necessary antiviral immune responses and interfere with normal tissue repair. Timing of intervention is critical-early inhibition may impair host defense, while delayed treatment might be ineffective after tissue damage has already occurred. Additionally, prolonged Hippo pathway inhibition could potentially increase cancer risk, while complete NLRP3 suppression might enhance susceptibility to secondary bacterial infections. To address these concerns, developing lung-specific delivery systems or temporally controlled interventions could help mitigate these risks while maintaining therapeutic benefits.

However, our study has some limitations. First, the limitation of our relatively small clinical sample size may affect the generalizability of our findings to broader patient populations. Future studies with larger cohorts and more diverse patient demographics would strengthen our conclusions and potentially reveal additional insights into the role of the Hippo pathway in viral pneumonia. Second, while our study primarily utilized poly(I:C) as a viral mimetic to induce lung injury, it remains to be determined whether our findings are specific to poly(I:C)-induced responses or can be generalized to various viral infections. Although poly(I:C) effectively mimics double-stranded RNA viruses and is widely used in experimental models, different viruses may engage distinct molecular mechanisms. Further studies with specific viral pathogens such as influenza, respiratory syncytial virus, and coronaviruses are needed to confirm the broader applicability of our findings. Third, the pharmacological inhibitors LATS-IN-1 targeting the Hippo pathway components, may have off-target effects that could influence our results. Future studies using additional Hippo pathway inhibitors, or using CRISPR-Cas9 gene editing would provide more comprehensive evidence for the specific roles of Hippo pathway in NETosis and viral lung injury. Fourth, it remains unclear whether other viruses also activate the Hippo pathway to promote NETosis via NLRP3 inflammasome-mediated IL-1β secretion. Additionally, in vivo studies using knockout mice should be performed to explore the underlying molecular mechanisms.

In conclusion, we identified a crucial function of NETosis and Hippo pathways in virus-induced lung injury. NETosis aggravates virus-induced lung injury and is increased in viral pneumonia by regulating the Hippo pathway via the NLRP3/IL-1β axis. Drug screening for Hippo and NLRP3/IL-1β pathways may suppress excessive inflammatory responses and intercept NETosis during viral infection.

## Materials and methods

### Clinical sample

Our study involved a total of 20 participants, which included 10 severe viral pneumonia patients and 10 healthy volunteers, all sourced from the Intensive Care Unit of the Affiliated Tumor Hospital of Guangxi Medical University (Supplemental Table 1). The pneumonia patients were diagnosed based on acute respiratory infection symptoms and confirmed viral infection through direct immunofluorescence antigen detection, real-time fluorescence PCR nucleic acid detection, or pathogen metagenomic next-generation sequencing. A Cycle threshold (Ct) value of ≤35 was considered positive for viral presence, with values between 35 and 40 considered weakly positive. Our inclusion criteria for pneumonia diagnosis required fever, cough, wheezing, accelerated breathing, and wet rales on lung auscultation, or chest imaging indicative of pneumonia. Severe pneumonia was diagnosed based on one primary criterion or three or more secondary criteria, including the need for invasive mechanical ventilation or vasoconstrictor treatment for septic shock. All participants or their legally authorized representatives provided written informed consent prior to enrollment in the study. For patients with severe viral pneumonia who were unable to provide consent due to their critical condition, informed consent was obtained from a legally authorized surrogate, such as a family member or guardian, following institutional ethical guidelines. The consent process was conducted by trained clinical investigators who provided verbal and written explanations of the study’s purpose, procedures, potential risks, and benefits in a clear and comprehensible manner. The study protocol was approved by the Ethics Committee of the Guangxi Medical University Tumor Hospital (approval number 2023-3-7), adhering to the guidelines of the Declaration of Helsinki. Peripheral blood was collected from both groups at 8 AM, with neutrophils isolated using density gradient centrifugation and serum retained and stored at −80 °C for further analysis.

### Mice

All animal experiments were conducted using wild-type male C57BL/6 J mice, aged 6–8 weeks and weighing approximately 25 ± 5 g, obtained from the Animal Center of Guangxi Medical University (Nanning, China). Animals had not been previously used in any other experiments. They were housed in a controlled environment with filtered air, free access to food and water, a temperature of 20–25 °C, and relative humidity of 50–70%. Animals were randomly assigned to experimental groups using a computer-generated randomization sequence. Inclusion and exclusion criteria were pre-established based on animal health and viability, and no animals or samples were excluded from analysis. Anesthesia was administered using ketamine hydrochloride and xylazine to minimize discomfort. All procedures involving animals were approved by the Institutional Animal Care and Use Committee (IACUC) of Guangxi Medical University and conducted in accordance with the Guide for Regulation and Administration of Laboratory Animals of the People’s Republic of China. The study also complied with the ARRIVE guidelines (PLoS Biol. 8(6), e1000412, 2010). Blinding was not performed during experimentation or outcome assessment.

### Cell

RAW 264.7 cells were purchased from ATCC and were authenticated using short tandem repeat (STR) profiling within the past 12 months. The cells were tested and confirmed negative for mycoplasma contamination before use. Primary alveolar macrophages (AMs) were generated from wild-type C57BL/6 mice with or without poly(I:C) stimulation (HMW, tlrl-pic; InvivoGen, USA). Briefly, primary AMs were obtained from bronchoalveolar lavage fluid (BALF) after erythrocyte lysis. BALF was plated for 1 h, followed by thorough washing to remove unattached cells. Adherent cells were used as primary AMs [28]. RAW 264.7 cells and AMs were cultured in RMPI 1640 medium containing 10% fetal bovine serum (FBS) (10091148, Gibco, New Zealand), 20 mM HEPES, and 2 mM L-glutamine. Following this separation, AMs were further isolated using magnetic bead separation with CD14 MicroBeads (MiltenyiBiotec, Germany), targeting the CD14+ monocyte population, which is a standard practice for monocyte isolation due to its high specificity and efficiency. Approximately 95% of the harvested cells were alveolar macrophages, as confirmed by flow cytometry.

Neutrophils were extracted from ethylenediaminetetraacetic acid (EDTA) (E809069, Macklin, China)-anticoagulated entire blood collected from wild-type C57BL/6 mice with or without poly(I:C) stimulation using density gradient centrifugation. The entire blood was layered upon a density gradient comprising a lower layer of Histopaque®-1119 (11191, Sigma-Aldrich, Vienna, Austria) and an upper layer of Ficoll-Paque PLUS (17-1440-03, GE Healthcare, Uppsala, Sweden), followed by centrifugation at 700 × *g* lasting for 30 min. The fraction comprising polymorphonuclear cells was located above the erythrocyte pellet and was carefully gathered, then washed with 1 × Dulbecco’s phosphate-buffered saline (DPBS) (15575-020, Thermo Fisher Scientific, Vienna, Austria). Subsequently, these cells were resuspended in VersaLyse Lysing Solution (A09777, Beckman Coulter, Marseille, France) aimed at eliminating red blood cells. The purity of the neutrophil population was typically higher than 90% as evaluated via flow cytometry. Viability of the immunomagnetically isolated neutrophils was evaluated by flow cytometry using cell nucleic acid fluorescent dye, Sytox-Green. For flow cytometry, live single-cell suspensions at a concentration of 1 × 10^6^ cells/ml were first blocked with anti-mouse CD16/32 Fc receptor block followed by surface labeling of anti-CD45, anti-CD11b, and anti-Ly6G antibodies at room temperature for 20 min. Cells were then washed three times, resuspended in 1 ml of DPBS, and run on a cell analyzer. In the co-culture experiment, neutrophils isolated from peripheral blood of control mice were cultured with conditioned medium collected from RAW 264.7 cells that had been treated under different conditions (control, poly(I:C) stimulation, or poly(I:C) plus Lats-IN-1). Neutrophils were seeded at a concentration of 1 × 10^6^ cells/mL and co-cultured with the conditioned medium for 48 h. Following co-culture, neutrophils were collected for analysis of NETosis markers using immunofluorescence, Western blotting, and ELISA.

### Reagents administration

C57BL/6 mice were intranasally challenged with 5 mg/Kg high-molecular-weight poly(I:C) at a concentration of 1 mg/mL to induce ALI/ARDS [29]. This administration was performed under light anesthesia to ensure precise delivery and minimize stress to the animals. The mice were euthanized after the final treatment to collect serum, BALF, and lung tissues for further downstream examinations. In vitro, RAW 264.7 cells, primary AMs, or co-cultures of conditioned medium and neutrophils were exposed to poly(I:C) (20 µg/mL) stimulation for 48 h with or without interventions [30].

Hippo pathway inhibitor, Lats-IN-1 (MedChemExpress, USA), is a potent and ATP-competitive inhibitor of LATS1 and LATS2 kinases. The administration regimen for Lats-IN-1 involved a 10 mg/kg intraperitoneal injection daily for 3 days, initiated 24 h before and continued simultaneously with and 24 h after administration of Poly(I:C) [31]. Neutralizing IL-1β antibody (R&D Systems, Germany) were administered at a dose of 10 mg/kg via intraperitoneal injection daily for 2 days, timed concurrently with and 24 h following Poly(I:C) exposure. In vitro treatments included exposure of RAW 264.7 cell lines to 10 µM Lats-IN-1, and 5 µL/mL of neutralizing IL-1β antibody [32].

### Plasmids, small interfering RNAs (siRNAs), and transfection

All shRNAs used in this study were provided by Sangon (Shanghai, China), as listed in Supplemental Table 2. All procedures related to the experiment were carried out as per the guidelines provided by the manufacturer. The supplementary material holds the detailed methods.

### Measurement of pulmonary edema, permeability, and cytokines

The right upper lobe with excess water was eliminated using filter paper to ascertain its weight (W). The lung tissues were subjected to a drying process at 60 °C for 48 h to attain their dry weight (D). The calculation of the W/D ratio was used as a measurement index for pulmonary edema. An evaluation of changes in lung permeability was conducted by assessing total BALF protein using a BCA Protein Assay Kit (23225, Thermo Fisher Scientific, Waltham, MA, USA). Additionally, a hemocytometer was used to count total cell infiltration. Interleukin 1β (IL-1β), tumor necrosis factor ɑ (TNF-ɑ), dsDNA, LL-37, and granulocyte-macrophage colony-stimulating factor (GM-CSF) levels in cell culture supernatant, plasma, and BALF were measured using enzyme-linked immunosorbent assay kits (CUSABIO, Wuhan, China).

### Histologic study

The lower lobes of the right lung were preserved using 4% paraformaldehyde (30525-89-4; Sigma-Aldrich, AR, USA), and then encapsulated within the Tissue-Tek OCT compound (4583; Sakura, Tokyo, Japan). The pathological assessment of lung damage was independently evaluated by two authors on sections stained with hematoxylin and eosin, following criteria that had been reported earlier [33]. In order to analyze the accumulation of collagenous fibers in pulmonary fibrosis, the lung tissues were encased in paraffin and dyed using Masson’s stain, following the guidelines provided by the manufacturer.

### Measurement of mRNA expression

Total mRNA of the cells was extracted using TRIzol reagent (Thermo Fisher Scientific) following the guidelines listed by the manufacturer. The High-Capacity cDNA Reverse Transcription Kit (Applied Biosystems, 4368814) was used to prepare the cDNA, which was then quantified using the PowerUp™ SYBR™ Green Maste Mix (Applied Biosystems, A25742). The relative expression levels of mRNA were determined using the 2-△△ct cycle threshold method. The primer sequences of NLRP3 used were as follows: forward: 5′-GGAGCGGGAGCATGAACTCC-3′, reverse: 5′-GGAGCGGGAGCATGAACTCC-3′. The fold change, adjusted to GAPDH normalization, was utilized to illustrate the variances between groups.

### Immunoblotting

The left lower lung lobes were thoroughly mixed into a uniform solution using RIPA lysis buffer (20-188, Sigma-Aldrich, AR, USA). During this process, to prevent protein degradation and dephosphorylation, both a Protease Inhibitor Tablet (product number 11836170001 from Roche, located in Basel, Switzerland) and a PhosphoSTOP Phosphatase Inhibitor Tablet (product number 4906845001, also from Roche in Basel, Switzerland) were added. This homogenization was achieved with the aid of a mechanical tissue homogenizer. The samples underwent lysis for a duration of 30 min at an icy temperature, followed by centrifugation at 12,000*g*-force for 15 min. Following the measurement of protein concentrations by the bicinchoninic acid (BCA) assay, the obtained supernatants from the cell lysates were heated to 85 °C for a duration of 5 min with a loading buffer added. Between 50 and 75 micrograms of proteins were subjected to separation through SDS-polyacrylamide gel electrophoresis (PAGE) and subsequently transferred to polyvinylidene fluoride (PVDF) membranes. After blocking a 1-h incubation period at 22–25 °C with 5% nonfat milk, the membranes underwent an overnight incubation with primary antibodies (Supplemental Table 3) at a temperature of 4 °C. This was followed by a 1-h incubation at room temperature with secondary antibodies (Abcam, Cambridge, UK) conjugated with horseradish peroxidase. Band intensities corresponding to different proteins were quantified from digitized films through the employment of an Odyssey® CLX imaging system (LI-COR, USA).

### Immunostaining

Air-dried for half an hour, the frozen tissue samples, cellular suspensions, or sheets of adherent cells were then stabilized with a 3.7% solution of paraformaldehyde for a quarter of an hour, followed by a chilling immersion in undiluted methanol for another 15 min. The slides underwent a blocking process using a solution of phosphate-buffered saline mixed with 3% goat serum (16210064, Gibco, CA, USA), 3% bovine serum albumin (SRE0096, Sigma-Aldrich, AR, USA), 0.2% Triton X-100 (Sigma-Aldrich, Arkansas, USA), and 0.02% NaN_3_ (S2002, Sigma-Aldrich, Arkansas, USA). Subsequently, they were treated with both primary antibodies and appropriate secondary antibodies. Comprehensive details regarding both the primary and secondary antibodies are provided in Supplemental Table 4. The specimens underwent a treatment process using ProLong®Gold Antifade Reagent containing 4’, 6-diamidino-2-phenylindole (DAPI) (8961S, CST, Massachusetts, USA). Then they were examined using multiplex confocal microscopy with a LSM980 microscope from Zeiss, located in Germany.

### Transcriptomics and processing of raw sequence data

Transcriptomes were conducted utilizing the Visium system from 10 × Genomics. Briefly, 10 mm fresh-frozen mouse lung sections, with or without poly(I:C) stimulation, were embedded in OCT and mounted on Visium slides, and the sections underwent a permeabilization procedure for 30 min to facilitate the release of mRNAs. These mRNAs subsequently adhered to the spatially barcoded oligonucleotides located on the underlying spots. Following this, a reverse transcription process was executed as per the manufacturer’s protocol. cDNA libraries were sequenced using the Illumina NextSeq 2000 system with a sequencing depth of over 50,000 reads for each spot, producing more than 400 million reads for each section. The software Spaceranger, at version 3. 1.0 by 10 × Genomics, performed alignment of individual spots from the Visium transcriptomics slides to the reference data of the GRCh38 mouse genome, resulting in the acquisition of raw counts (Supplemental Table 5). The data representing the expression patterns of the selected genes were submitted to the Database for Annotation, Visualization, and Integrated Discovery (DAVID) to conduct a Gene Ontology (GO) enrichment investigation, which encompasses the analysis of biological activities, cellular constituents, and molecular functionalities. All hub genes underwent analysis using DAVID for GO enrichment and KEGG pathway investigation, with counts > 5 and *p* < 0.01. To assess the interactive networks connecting all targeted genes, the STRING database was employed.

We identified differentially expressed genes (DEGs) between the normal group (Control, *n* = 6) and the viral lung injury group (Poly I:C, *n* = 6) using the R package “limma (version 3.50.0)” [33]. The screening criteria were *p* < 0.05 and |log2FC | > 1, and these DEGs were used for subsequent analyses. The “pheatmap” R package was employed to generate heatmaps, with clustering performed using Euclidean distance and hierarchical clustering methods.

The R package “clusterProfiler (version 4.2.2)” [34] was applied to conduct GO and KEGG enrichment analyses on the DEGs between all groups, with a significance threshold for pathway identification set at *p* < 0.05. Additionally, Gene Set Enrichment Analysis (GSEA) was performed using the “clusterProfiler (version 4.2.2)” R package, ranking all genes based on their log2Fold Change values and conducting 1,000 gene set permutations. The reference gene set used for GSEA was the KEGG database containing all mouse pathway gene sets [35]. Gene sets with an adjusted *p*-value < 0.05 were considered significantly enriched.

The protein-protein interaction (PPI) network was constructed using the Search Tool for the Retrieval of Interacting Genes (STRING) online database, with protein pairs having an interaction score greater than 0.7 being selected [36]. The PPI network was then visualized using Cytoscape software for better presentation [37]. The top 100 genes from all 12 CytoHubba algorithms were extracted, and their intersection was taken [38]. The genes obtained from this intersection were considered to be involved in the differences between groups. The 12 CytoHubba algorithms used here include: Betweenness, Stress, Radiality, Eccentricity, node connect degree (Degree), density of maximum neighborhood component (DMNC), edge percolated component (EPC), maximal clique centrality (MCC), node connect closeness (Closeness), maximum neighborhood component (MNC), ClusteringCoefficient, and BottleNeck.

### Statistical analysis

Typically, experiments conducted in vitro were replicated three times (except where noted differently), with results shown as the average value ± standard error of the mean, based on a minimum of three separate experiments. Two groups were compared using the Student’s t-test, while the one-way ANOVA with Tukey’s post-hoc test was utilized for comparing more than two groups. Statistical significance was established when *p*-values were less than 0.05. Statistical evaluations were carried out with the GraphPad Prism 9 software (GraphPad Software, San Diego, CA, USA).

## Supplementary information

Total study participant demographics and clinical information.

Small interfering RNAs Sequence.

Immunoblotting Primary Antibodies

Immunostaining Primary Antibodies

Gene counts and annotation of microarray data

Raw data of western blots

## Data Availability

All relevant data were within the manuscript and its supplemental files.
