# Supplementary material for: Hippo pathway and NLRP3-driven NETosis in macrophages: Mechanisms of viral pneumoniaaggravation
Source: Cell Death Discov. 2025 Jul 14;11:323. doi: 10.1038/s41420-025-02556-z (PMC12260020; doi:10.1038/s41420-025-02556-z)
Supplement: Supplementary file 1 — Total study participant demographics and clinical information. [file 41420_2025_2556_MOESM1_ESM.docx]

**Supplemental Table 1.** Total study participant demographics and clinical information

| Characteristics | Pneumonia | Healthy |
| --- | --- | --- |
| n | 10 | 10 |
| Age, mean ± sd | 67.1 ± 11.628 | 55.5 ± 8.4886 |
| Sex (M/F), n (%) |  |  |
| M | 5 (25%) | 6 (30%) |
| F | 5 (25%) | 4 (20%) |
| Types of infecton, n (%) |  |  |
| SARS-CoV-2 | 10 (50%) | 0 (0%) |
| Severe of pneumonia, n (%) |  |  |
| severe | 10 (50%) | 0 (0%) |
| Invasive mechanical ventilation(Y/N), n (%) |  |  |
| Y | 10 (50%) | 0 (0%) |
| N | 0 (0%) | 10 (50%) |
| Respiratory System(Y/N), n (%) |  |  |
| Y | 10 (50%) | 0 (0%) |
| N | 0 (0%) | 10 (50%) |
| hypertension(Y/N), n (%) |  |  |
| Y | 2 (10%) | 2 (10%) |
| N | 8 (40%) | 8 (40%) |
| Diabetes(Y/N), n (%) |  |  |
| Y | 2 (10%) | 1 (5%) |
| N | 8 (40%) | 9 (45%) |
| CardiovascularDisease(Y/N), n (%) |  |  |
| Y | 4 (20%) | 0 (0%) |
| N | 6 (30%) | 10 (50%) |
| Kidney Disease(Y/N), n (%) |  |  |
| N | 8 (40%) | 10 (50%) |
| Y | 2 (10%) | 0 (0%) |
