# Supplementary material for: Hippo pathway and NLRP3-driven NETosis in macrophages: Mechanisms of viral pneumoniaaggravation
Source: Cell Death Discov. 2025 Jul 14;11:323. doi: 10.1038/s41420-025-02556-z (PMC12260020; doi:10.1038/s41420-025-02556-z)
Supplement: Supplementary file 2 — Small interfering RNAs Sequence. [file 41420_2025_2556_MOESM2_ESM.docx]

**Supplemental Table 2.** Small interfering RNAs Sequence

| Gene | Sequence(5′-3′) |
| --- | --- |
| shNLRP3_1 | GCCTACAGTTGGGTGAAATTTCAAGAGAATTTCACCCAACTGTAGGCTTTTTT |
| shNLRP3_2 | CCAGGAGAGAACCTCTTATTTCAAGAGAATAAGAGGTTCTCTCCTGGTTTTTT |
| shNLRP3_3 | GGACCTCAGTGACAATACTTTCAAGAGAAGTATTGTCACTGAGGTCCTTTTTT |
