# Supplementary material for: Hippo pathway and NLRP3-driven NETosis in macrophages: Mechanisms of viral pneumoniaaggravation
Source: Cell Death Discov. 2025 Jul 14;11:323. doi: 10.1038/s41420-025-02556-z (PMC12260020; doi:10.1038/s41420-025-02556-z)
Supplement: Supplementary file 3 — Immunoblotting Primary Antibodies [file 41420_2025_2556_MOESM3_ESM.docx]

**Supplemental Table 3.** Immunoblotting Primary Antibodies

| Immunoblotting Primary Antibodies | | | | | |
| --- | --- | --- | --- | --- | --- |
| Antibodies | Origin | Catalog | Molecular Weight（KDa） | Host | Dilution |
| Anti-Histone H3 (citrulline R2 + R8 + R17) | ABCam | ab5103 | 17 | Rabbit | 1: 1000 |
| YAP1 | CST | 12395 | 70 | Mouse | 1: 2000 |
| Phospho-YAP1 (Ser397) | PTG | 29018-1-AP | 70 | Rabbit | 1: 1000 |
| MST1 | CST | 3682S | 60 | Rabbit | 1: 1000 |
| MST2 | CST | 3952 | 60 | Rabbit | 1: 1000 |
| Phospho-MST1 (Thr183)/MST2 (Thr180) | PTG | 80093-1-RR | 60 | Rabbit | 1: 1000 |
| LATS1 | CST | 3477S | 140 | Rabbit | 1: 1000 |
| Phospho-LATS1 (Thr1079) | Affinity | AF7169 | 140 | Rabbit | 1: 500 |
| IL-1β | ABCam | ab216995 | 15 | Rabbit | 1: 1000 |
| NLRP3 | CST | 15101 | 120 | Rabbit | 1: 1000 |
| α-tubulin | ABCam | ab7291 | 50 | Mouse | 1: 5000 |
| GAPDH | Servicebio | gb15002 | 37 | Mouse | 1: 2000 |
| Lamin B1 | Affinity | AF5161 | 68 | Rabbit | 1: 2000 |
