# Supplementary material for: Hippo pathway and NLRP3-driven NETosis in macrophages: Mechanisms of viral pneumoniaaggravation
Source: Cell Death Discov. 2025 Jul 14;11:323. doi: 10.1038/s41420-025-02556-z (PMC12260020; doi:10.1038/s41420-025-02556-z)
Supplement: Supplementary file 4 — Immunostaining Primary Antibodies [file 41420_2025_2556_MOESM4_ESM.docx]

**Supplemental Table 4.** Immunostaining Primary Antibodies

| Immunostaining Primary Antibodies | | | | |
| --- | --- | --- | --- | --- |
| Antibodies | Origin | Catalog | Host | Dilution |
| Anti-Histone H3 (citrulline R2 + R8 + R17) | ABCam | ab5103 | Rabbit | 1: 100 |
| Myeloperoxidase | Santa | sc-52707 | Mouse | 1: 100 |
| Myeloperoxidase | Biotechne | AF3667-SP | Goat | 1: 100 |
| LL-37 | Santa | sc-166770 | Mouse | 1: 100 |
| Neutrophil elastase | Invitrogen | PA5-115648 | Rabbit | 1: 200 |
| F4/80 | Servicebio | gb11027 | Rabbit | 1: 200 |
| Ly6G | Servicebio | gb11229 | Rabbit | 1: 200 |
| YAP1 | CST | 12395 | Mouse | 1: 100 |
