# Supplementary material for: Hippo pathway and NLRP3-driven NETosis in macrophages: Mechanisms of viral pneumoniaaggravation
Source: Cell Death Discov. 2025 Jul 14;11:323. doi: 10.1038/s41420-025-02556-z (PMC12260020; doi:10.1038/s41420-025-02556-z)
Supplement: Supplementary file 6 — Raw data of western blots [file 41420_2025_2556_MOESM6_ESM.docx]

**Figure 1D**

**
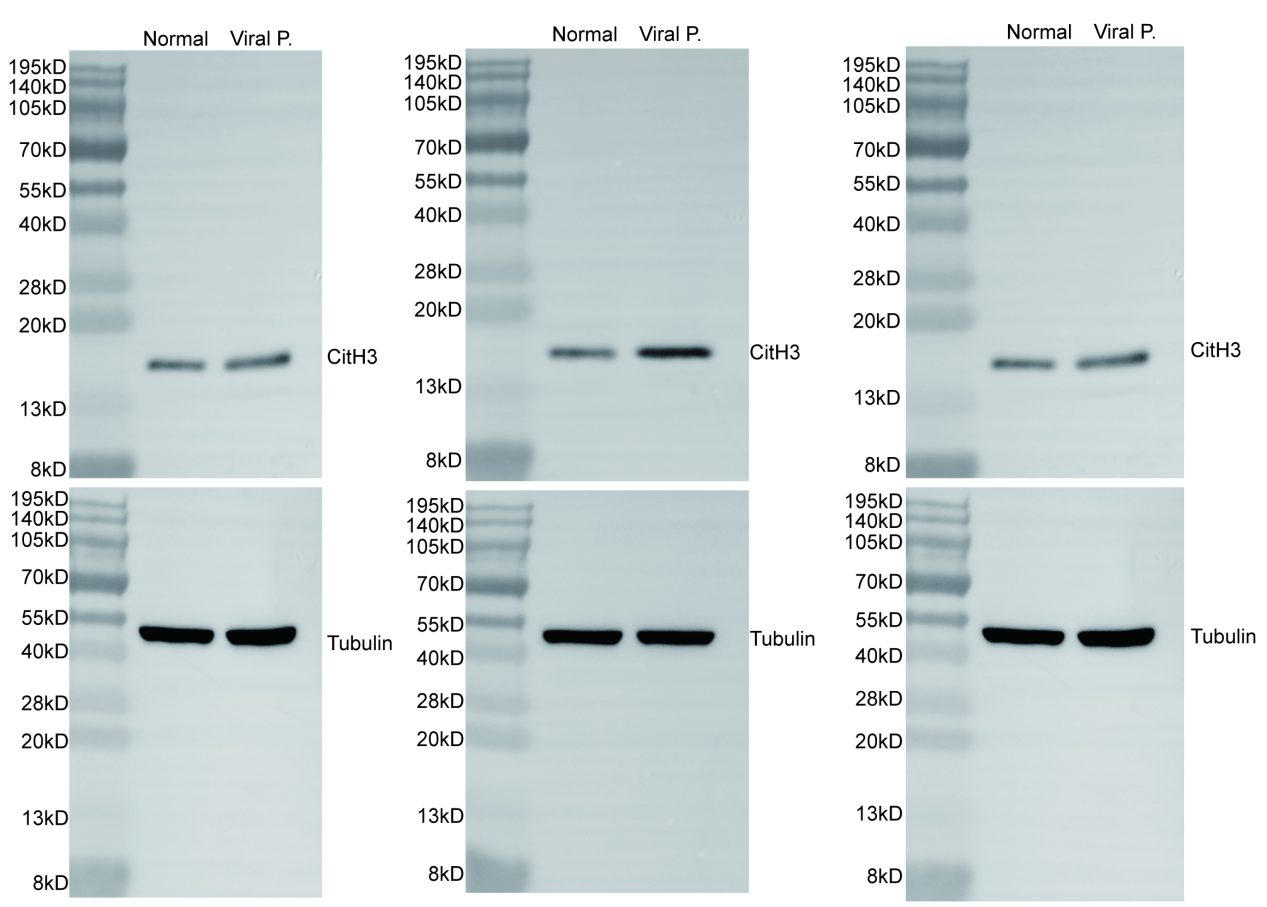
**

**Figure 2F**

**
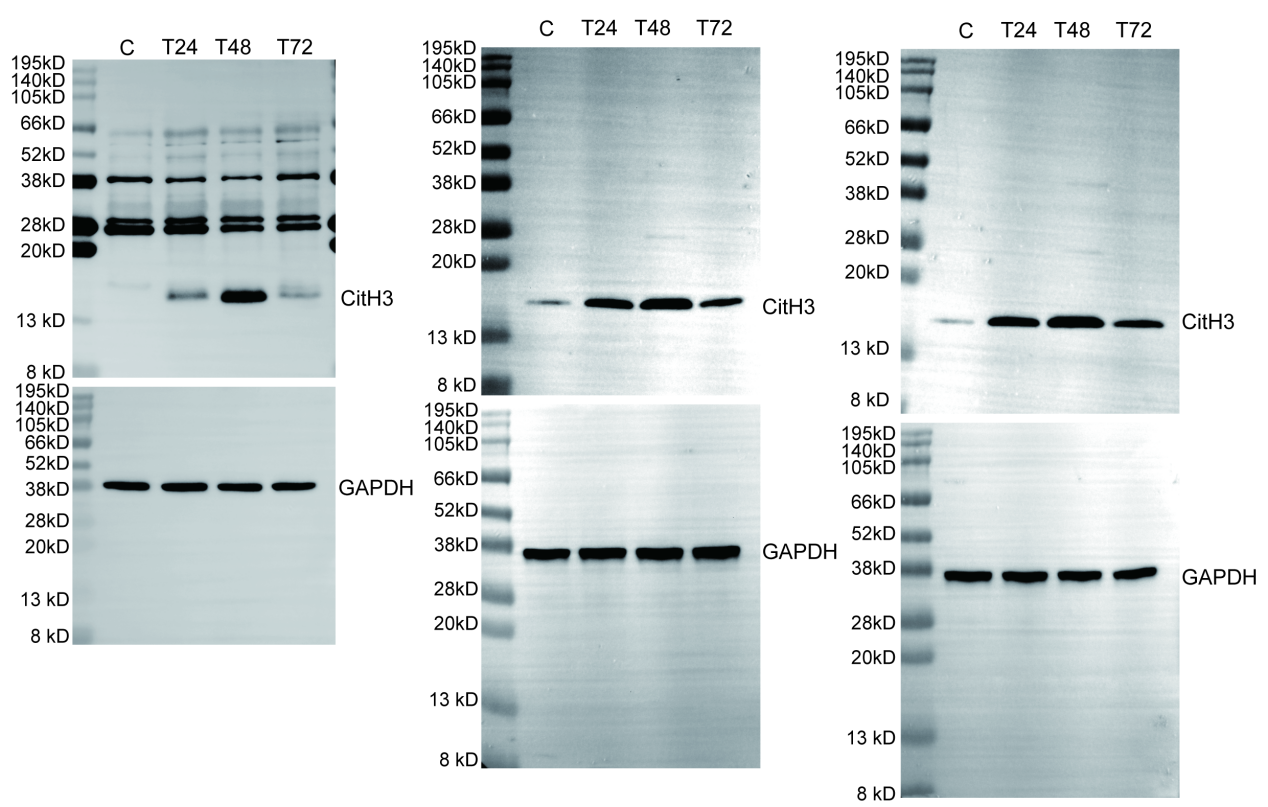
**

**Figure 3B**

**
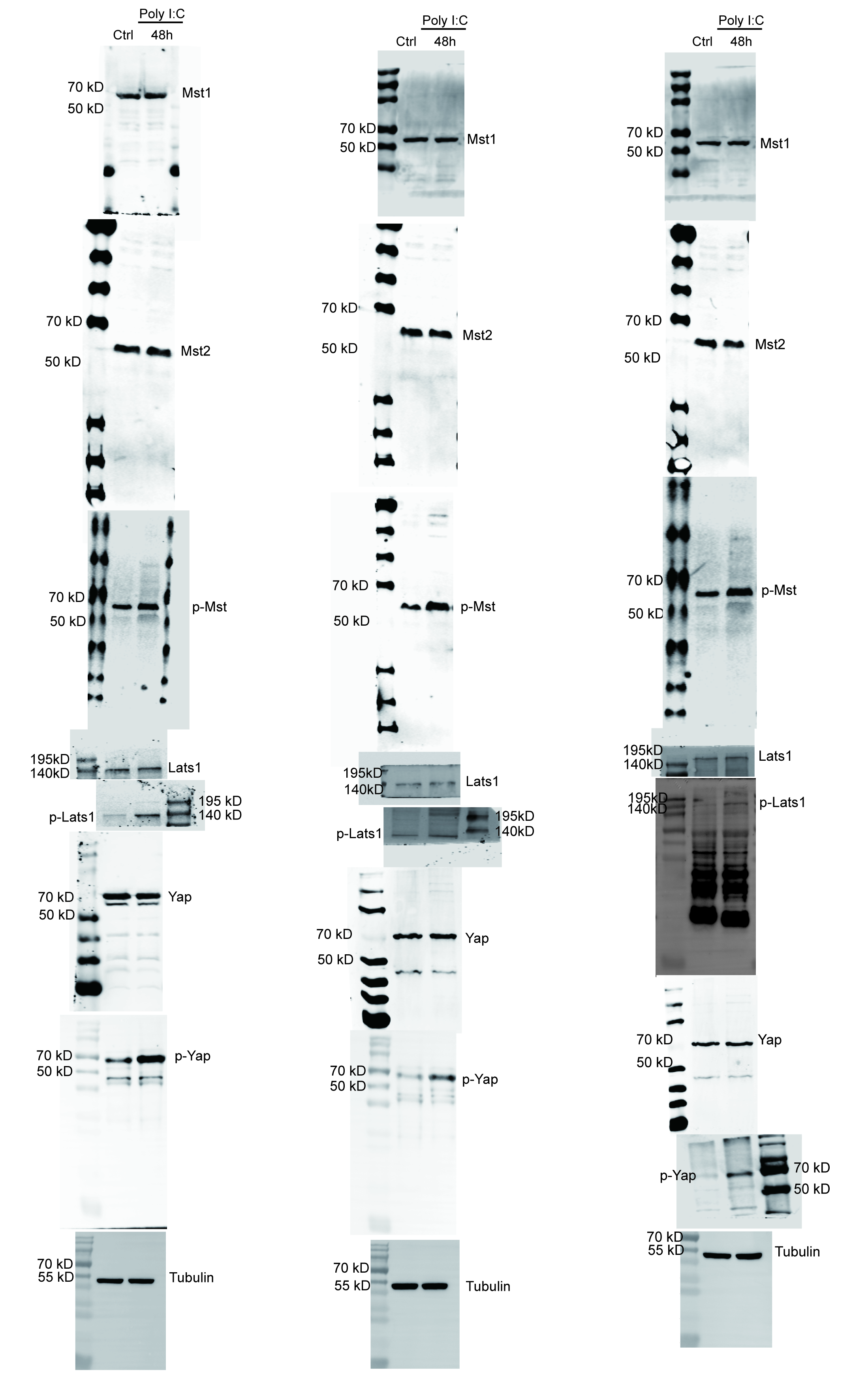
**

**Figure 3D**

**
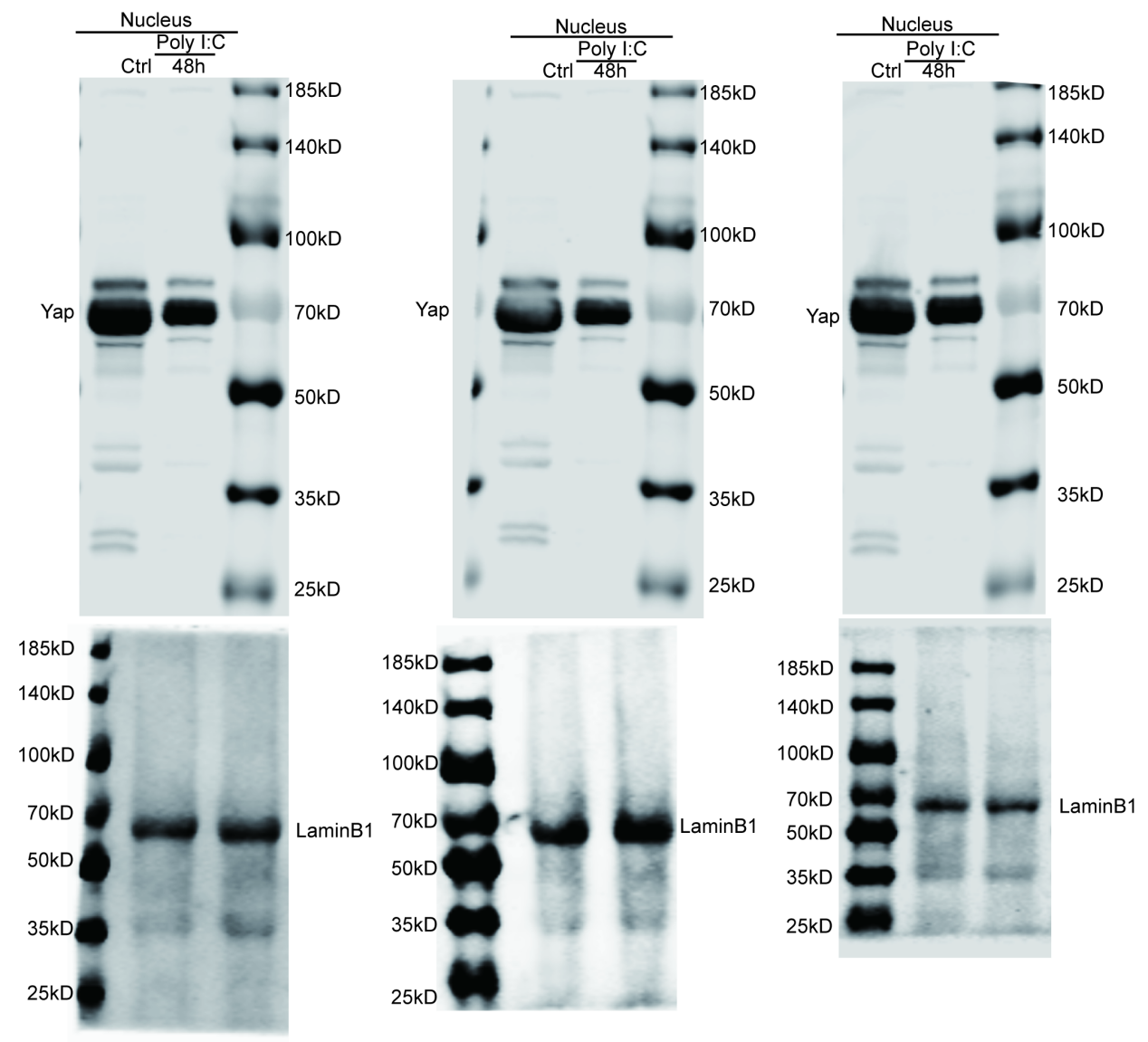
**

**Figure 3L**

**
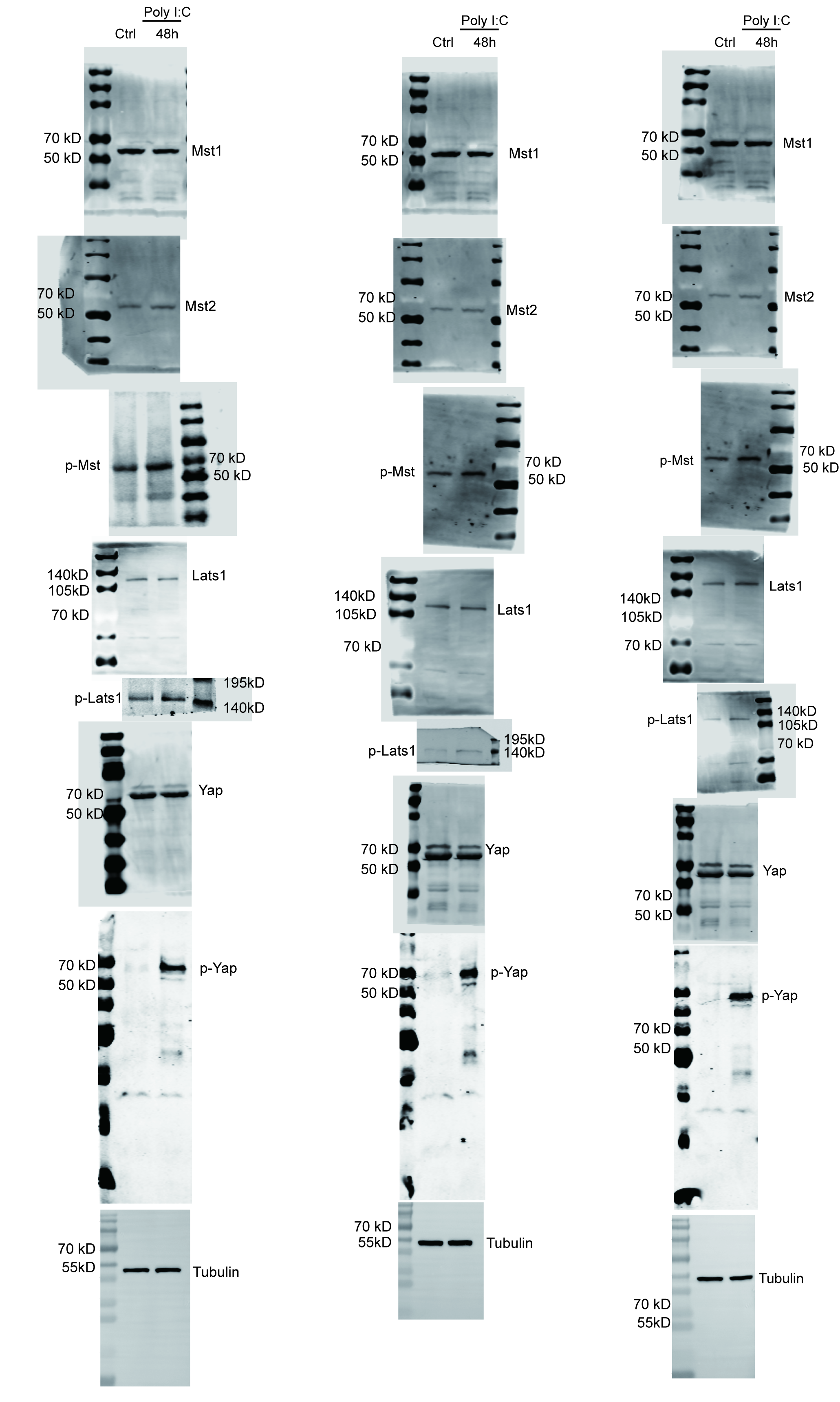
**

**Figure 3N**

**
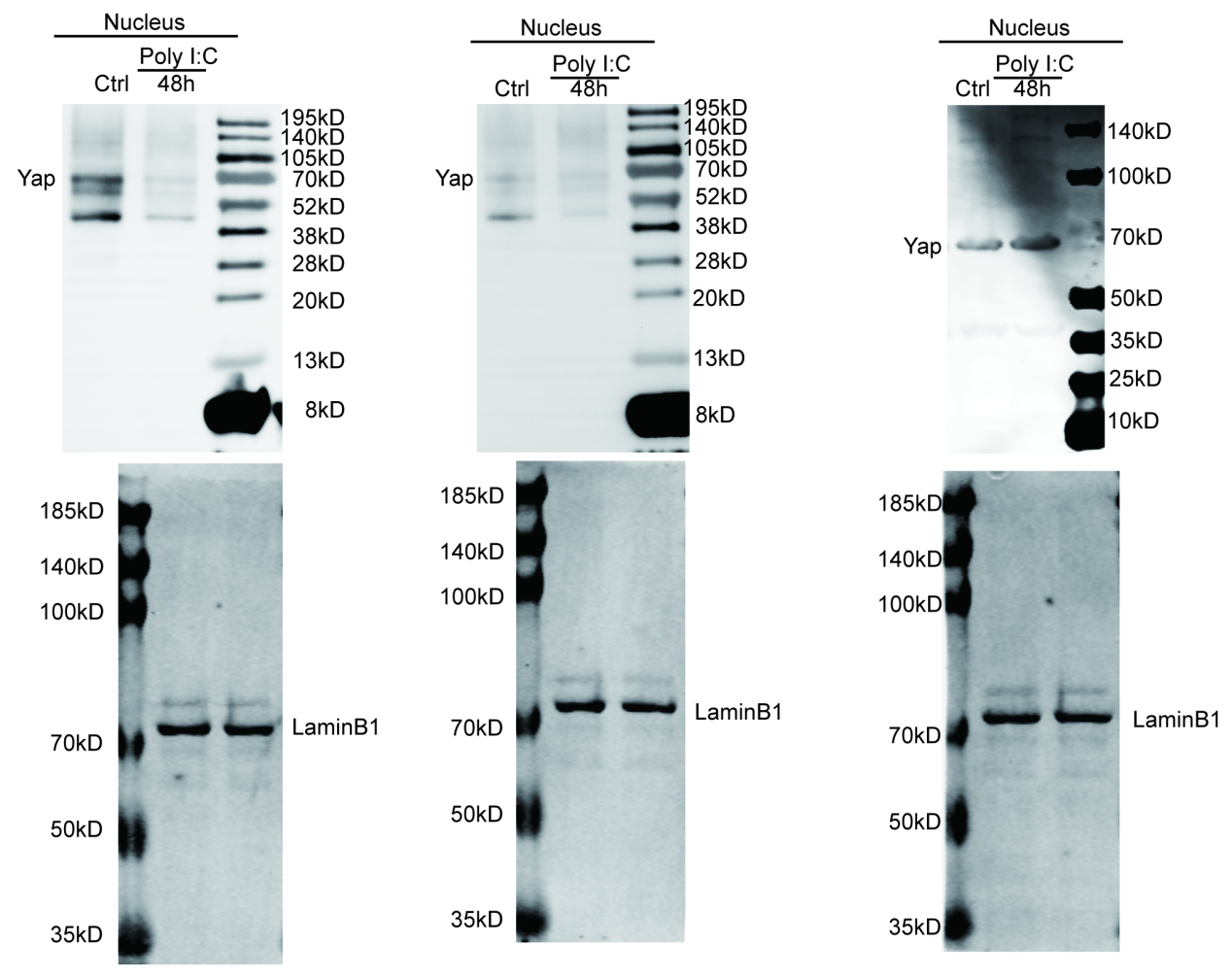
**

**Figure4A
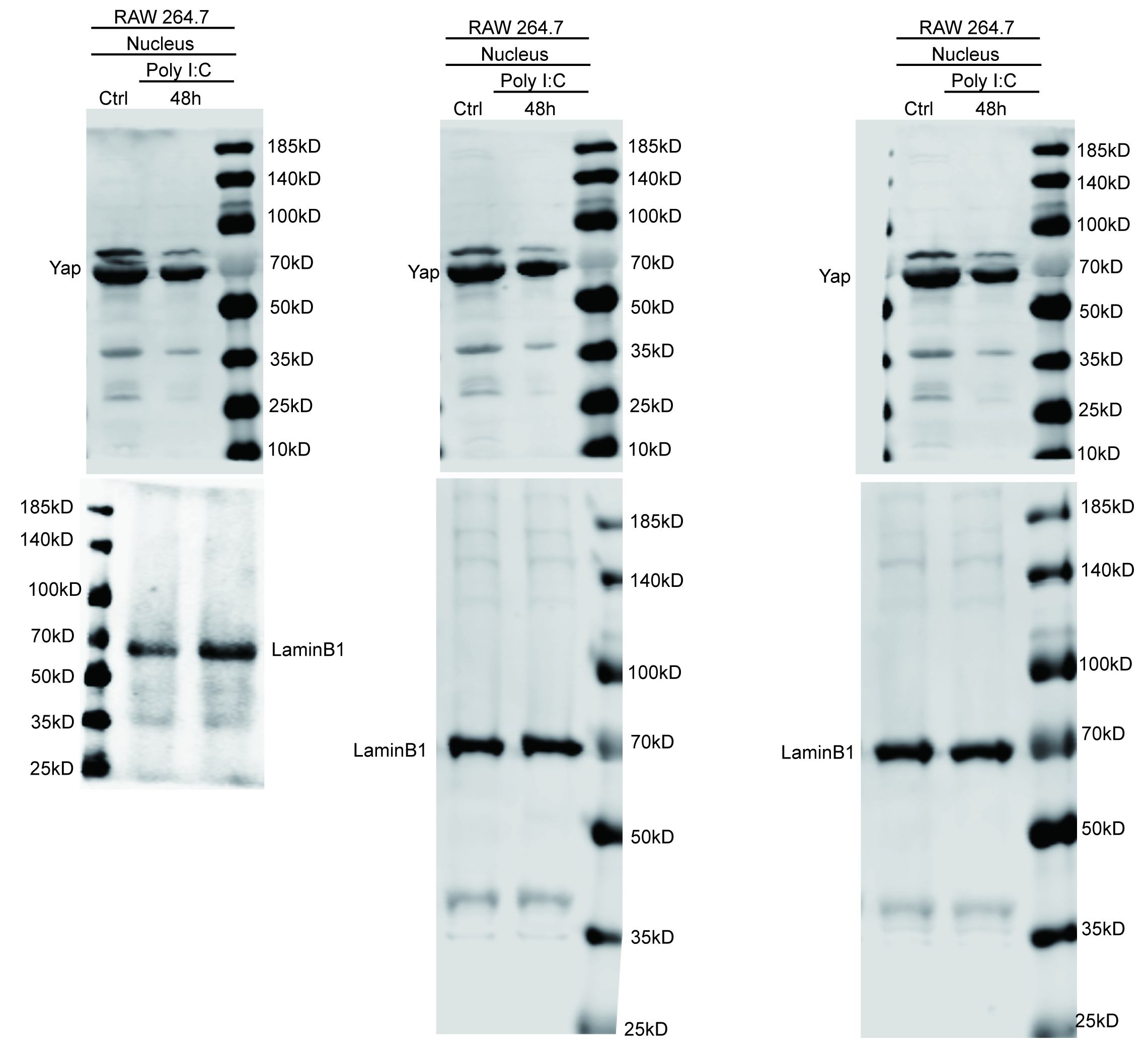
**

**Figure 4C**

**
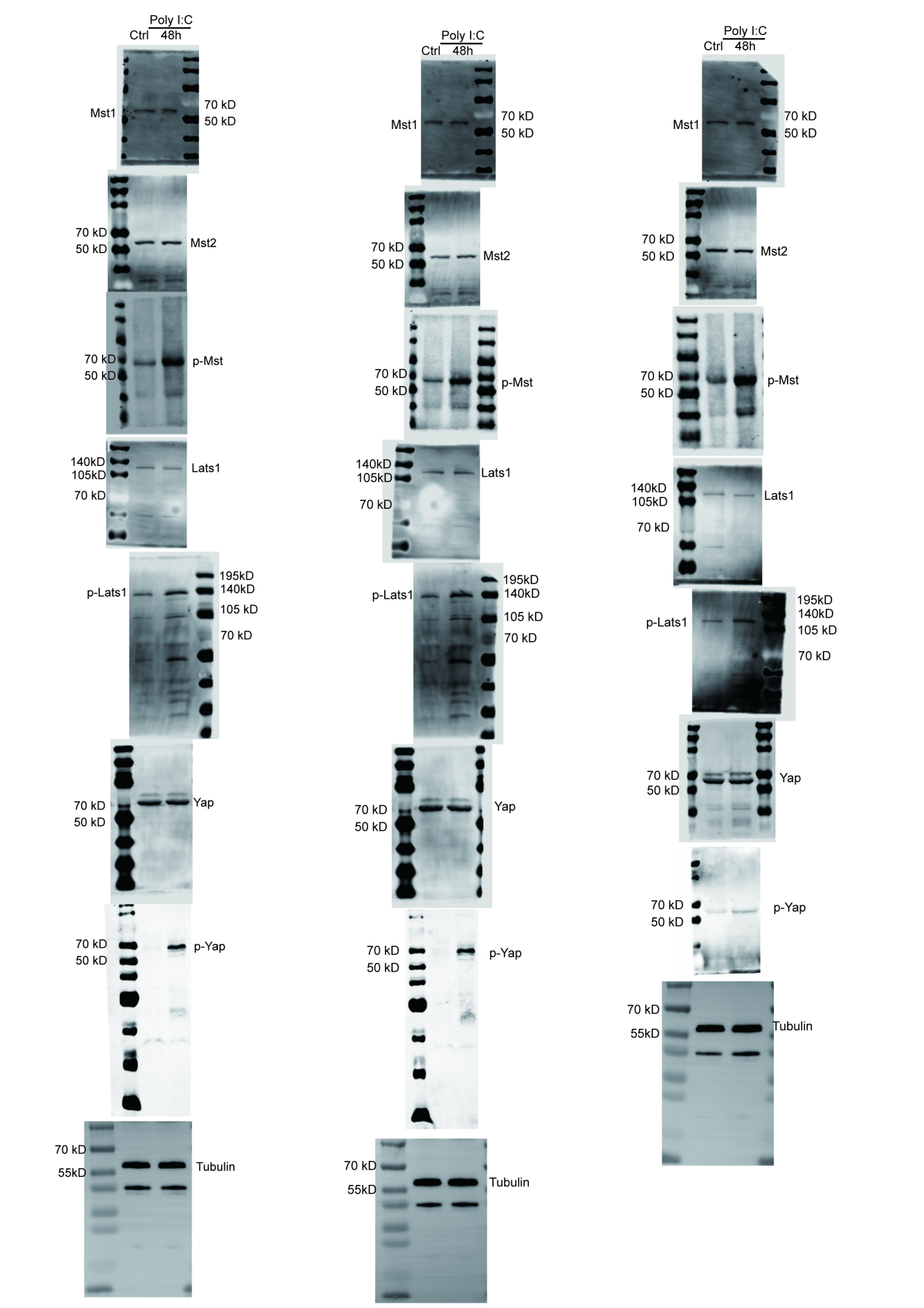
**

**Figure 4F**

**
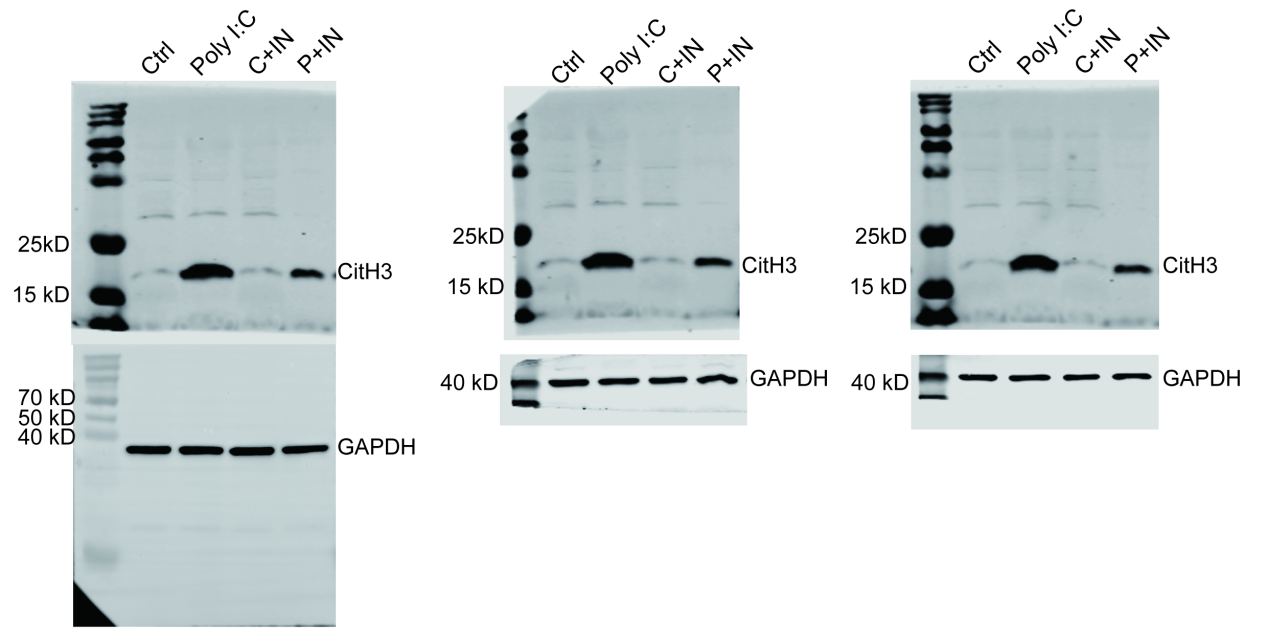
**

**Figure 5A**

**
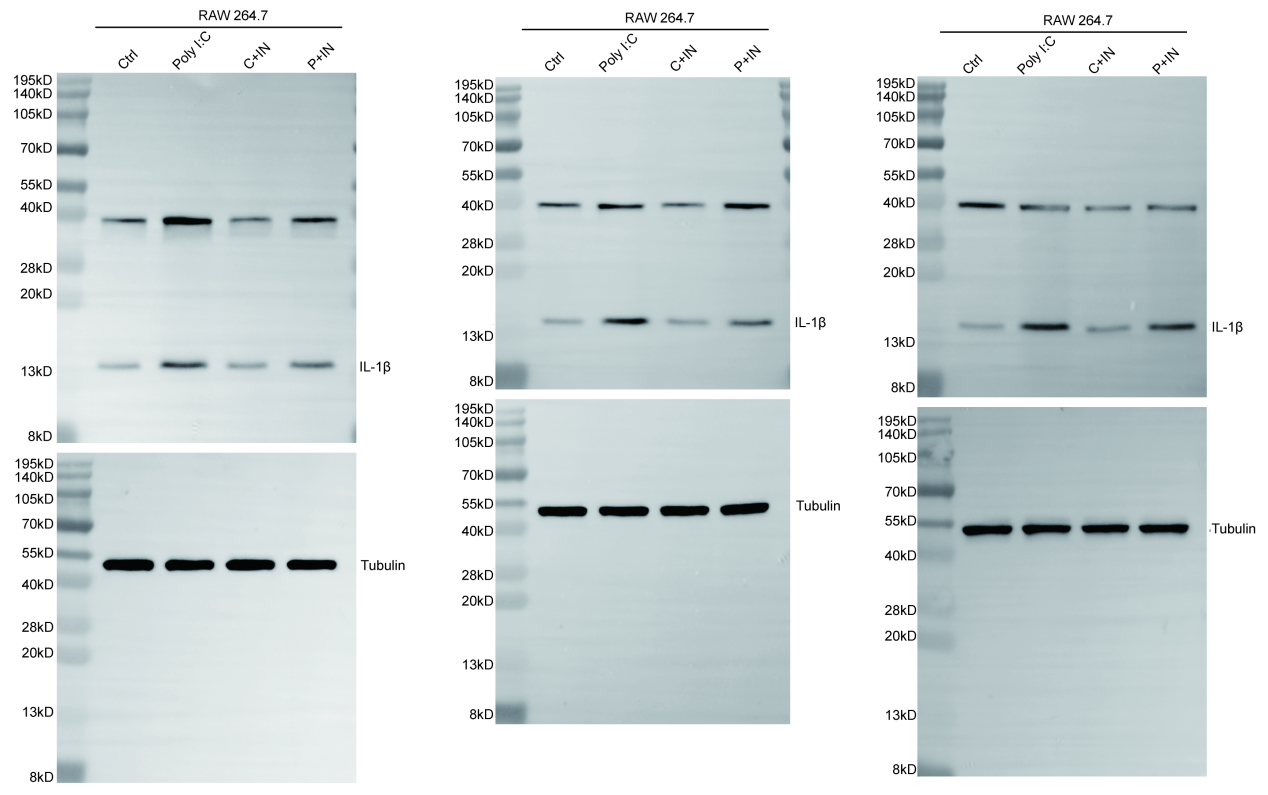
**

**Figure 5F**

**
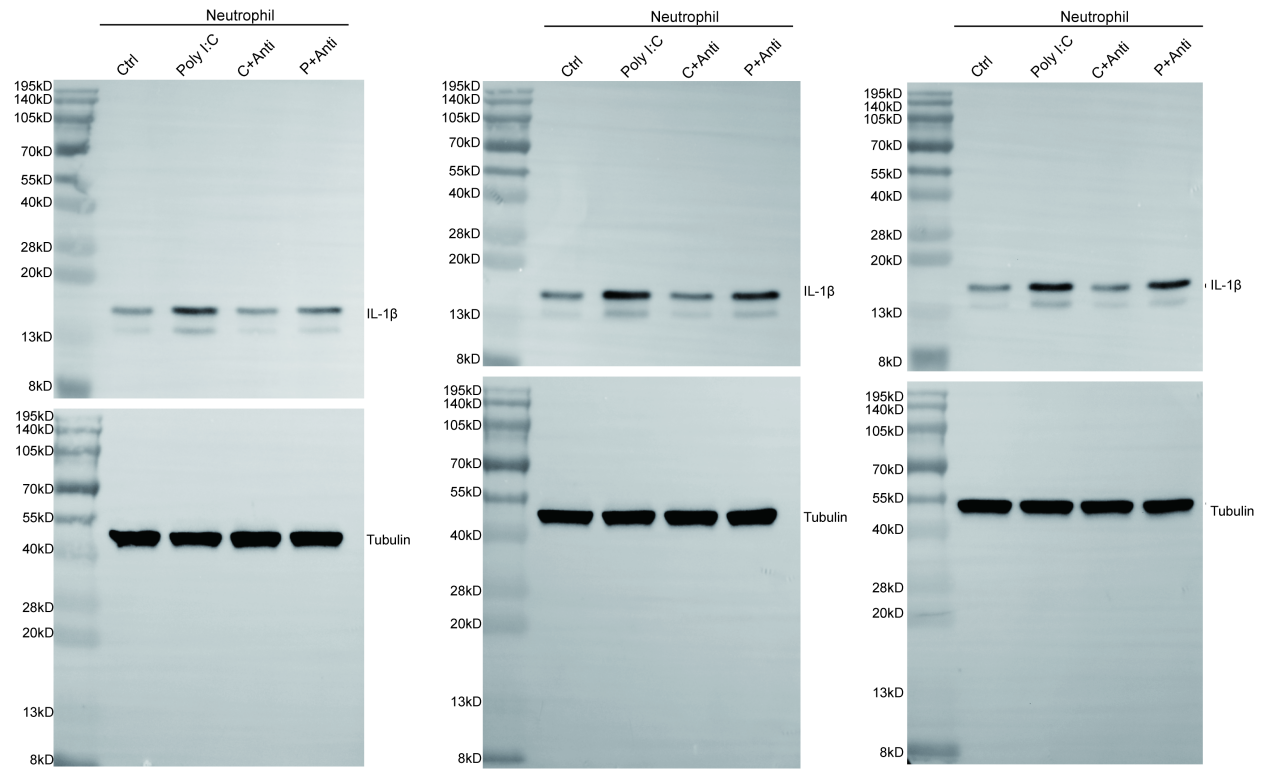
**

**Figure 6A**

**
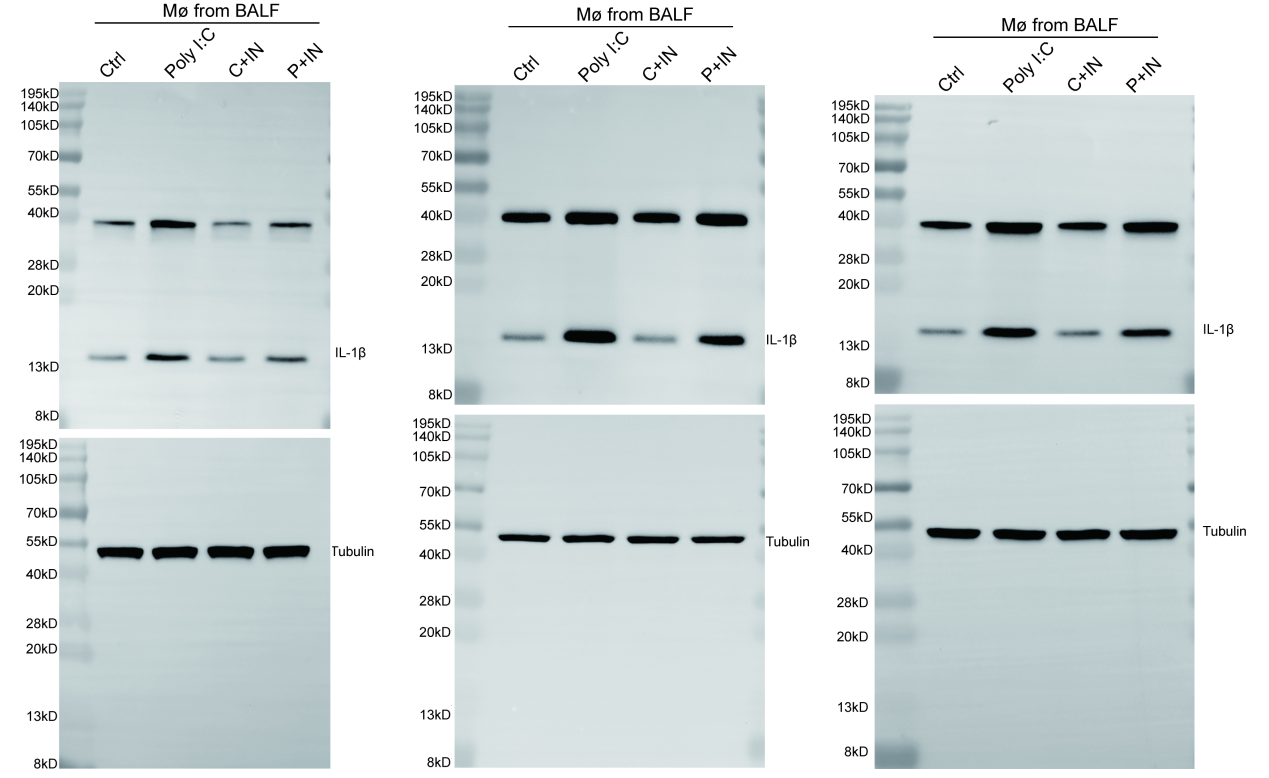
**

**Figure 6F**

**
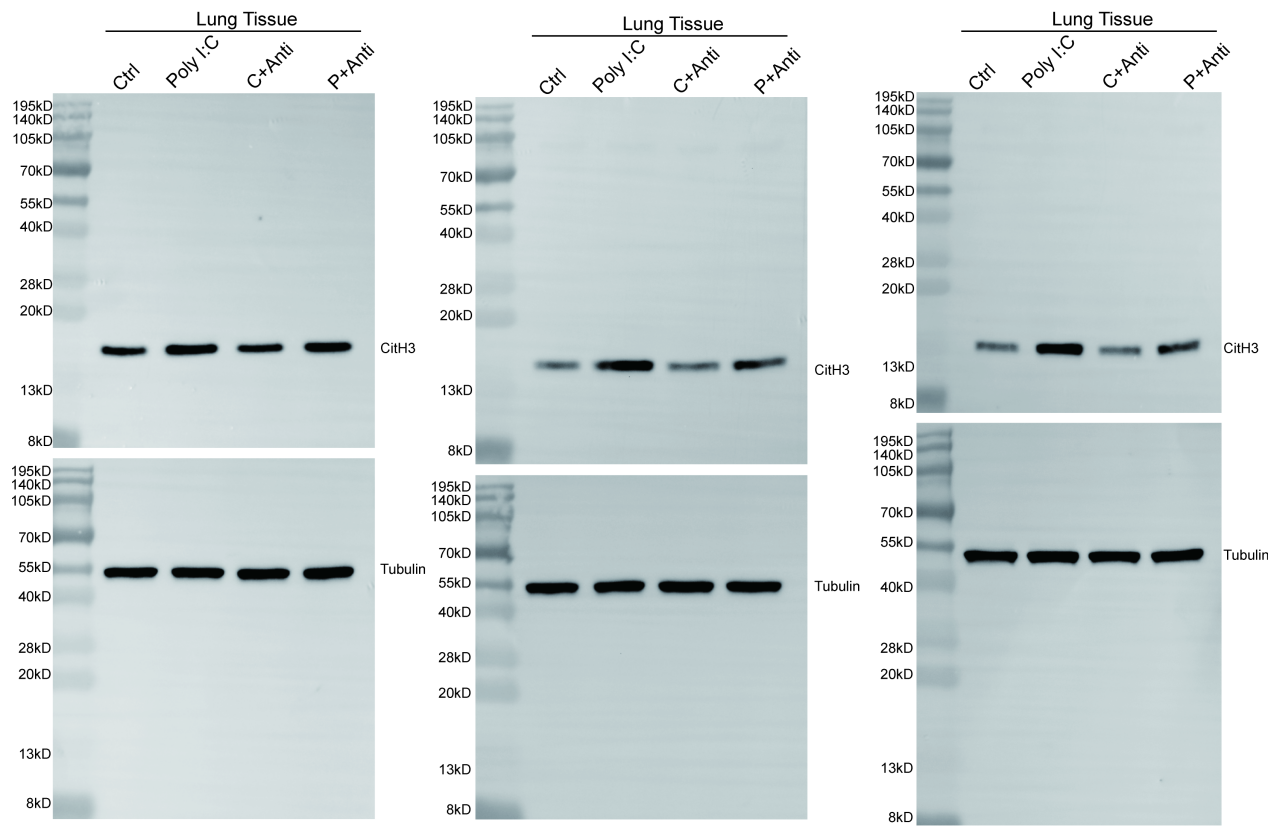
**

**Figure 8E**

**
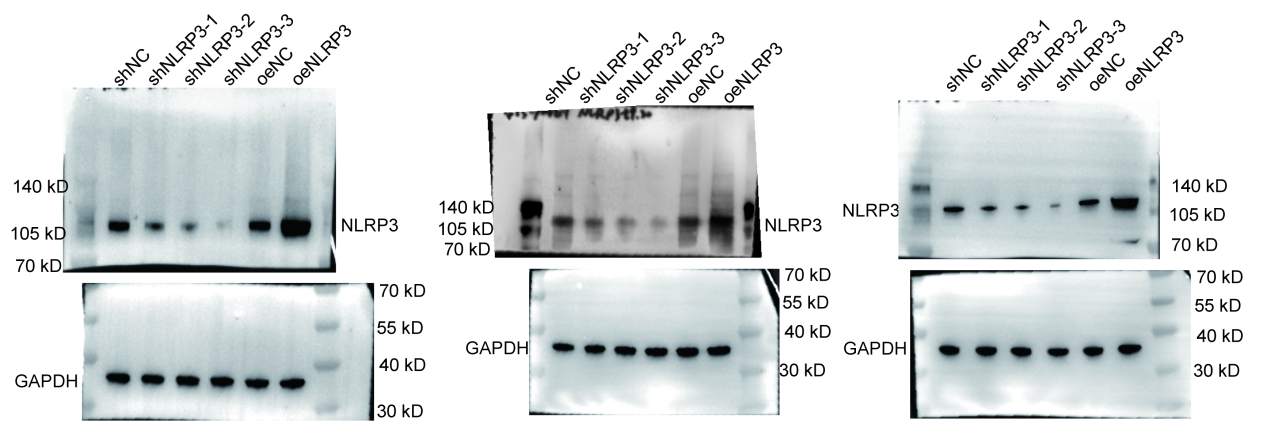
**

**Figure 8H**

**
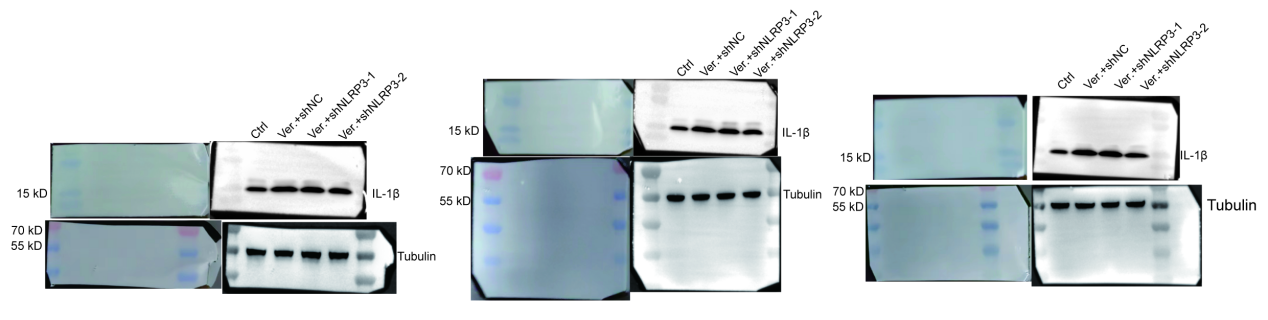
**

**Figure 9A**

**
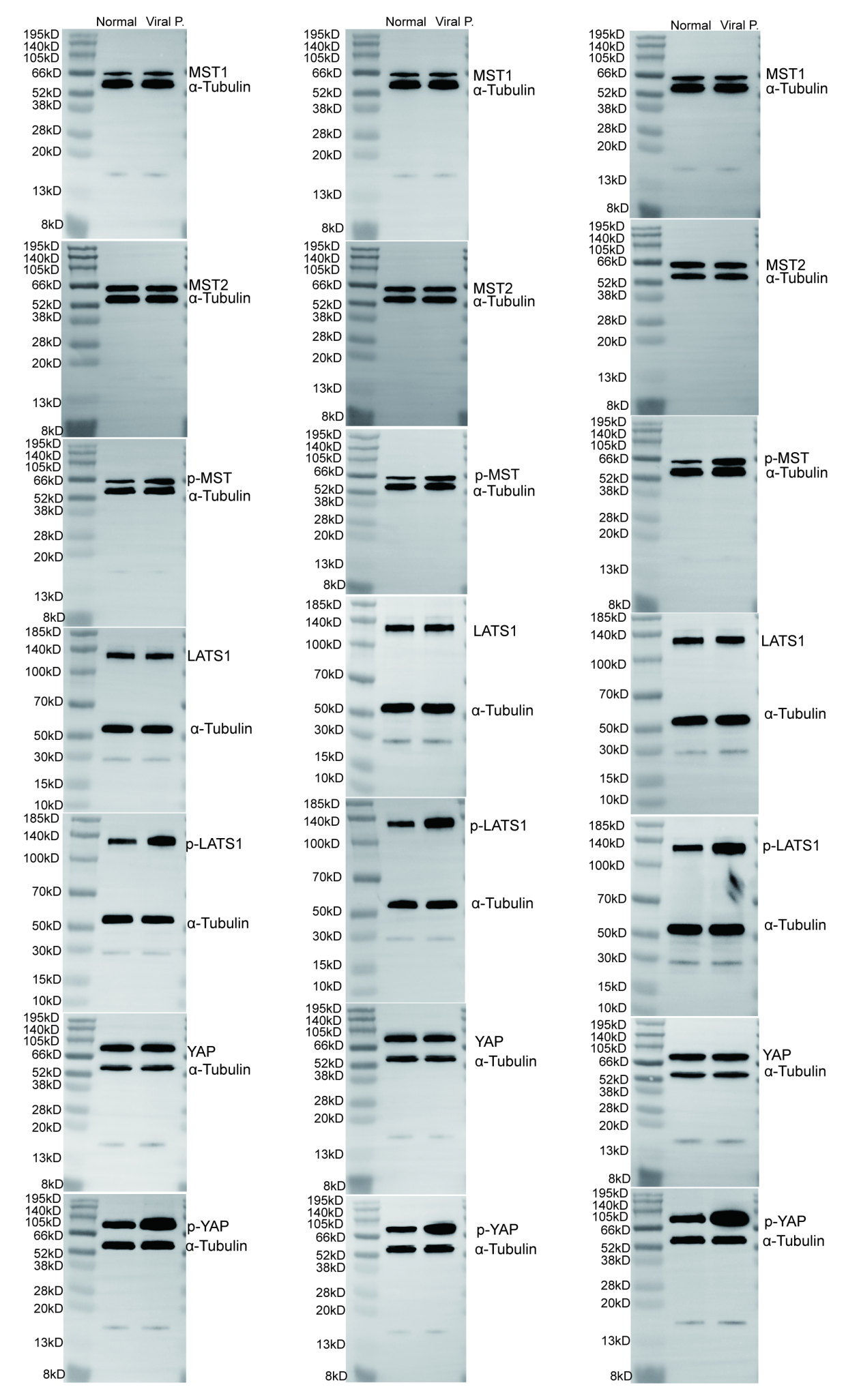
**

**Figure 9C**

**
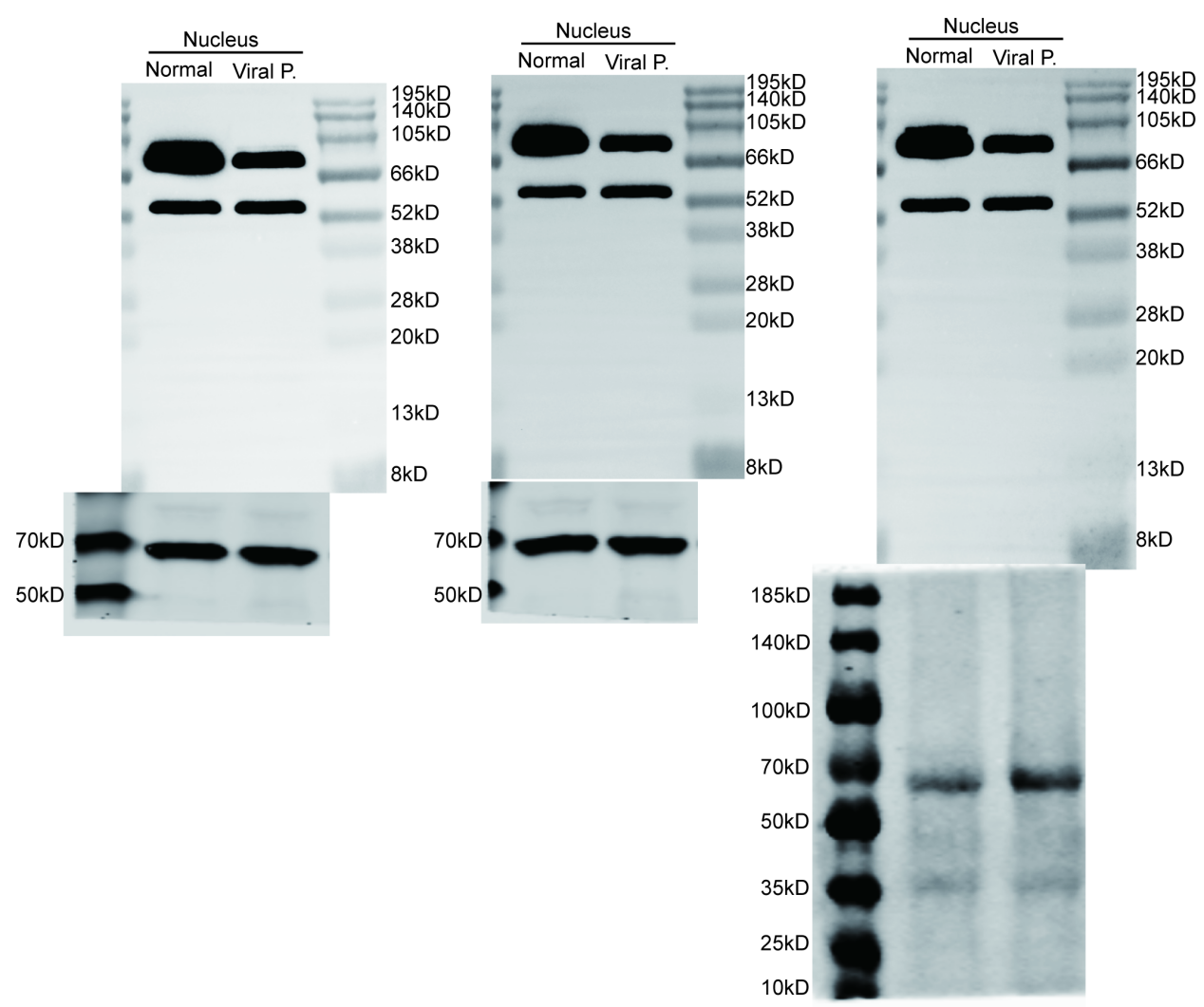
**

**Figure 9E**

**
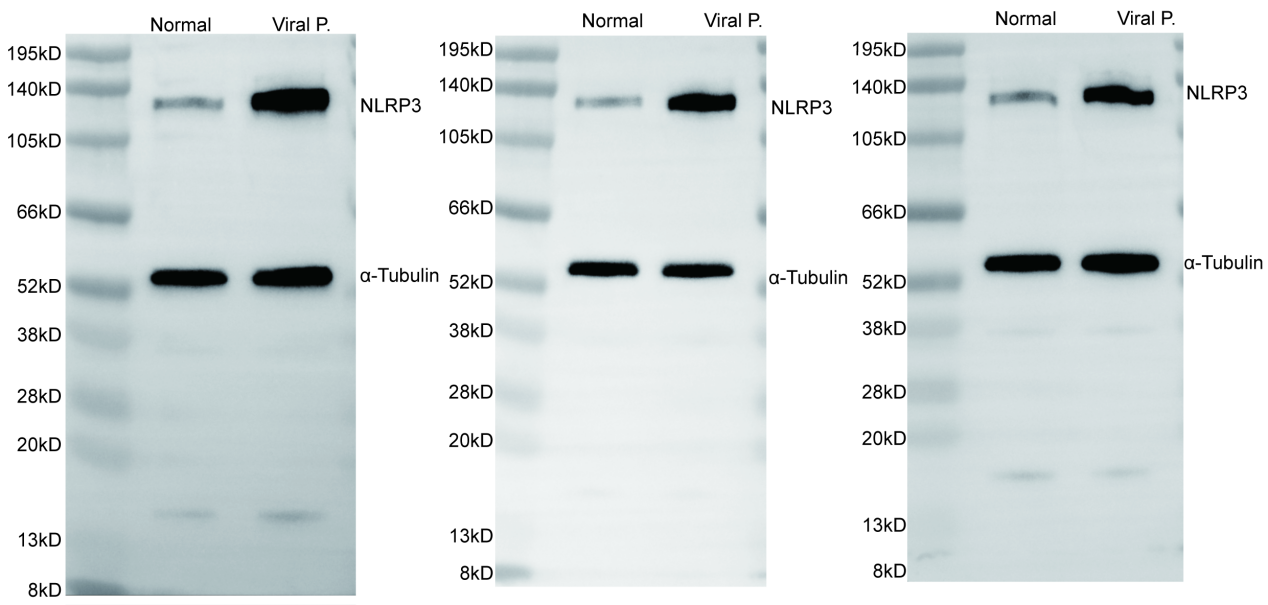
**

**Figure 9G**

**
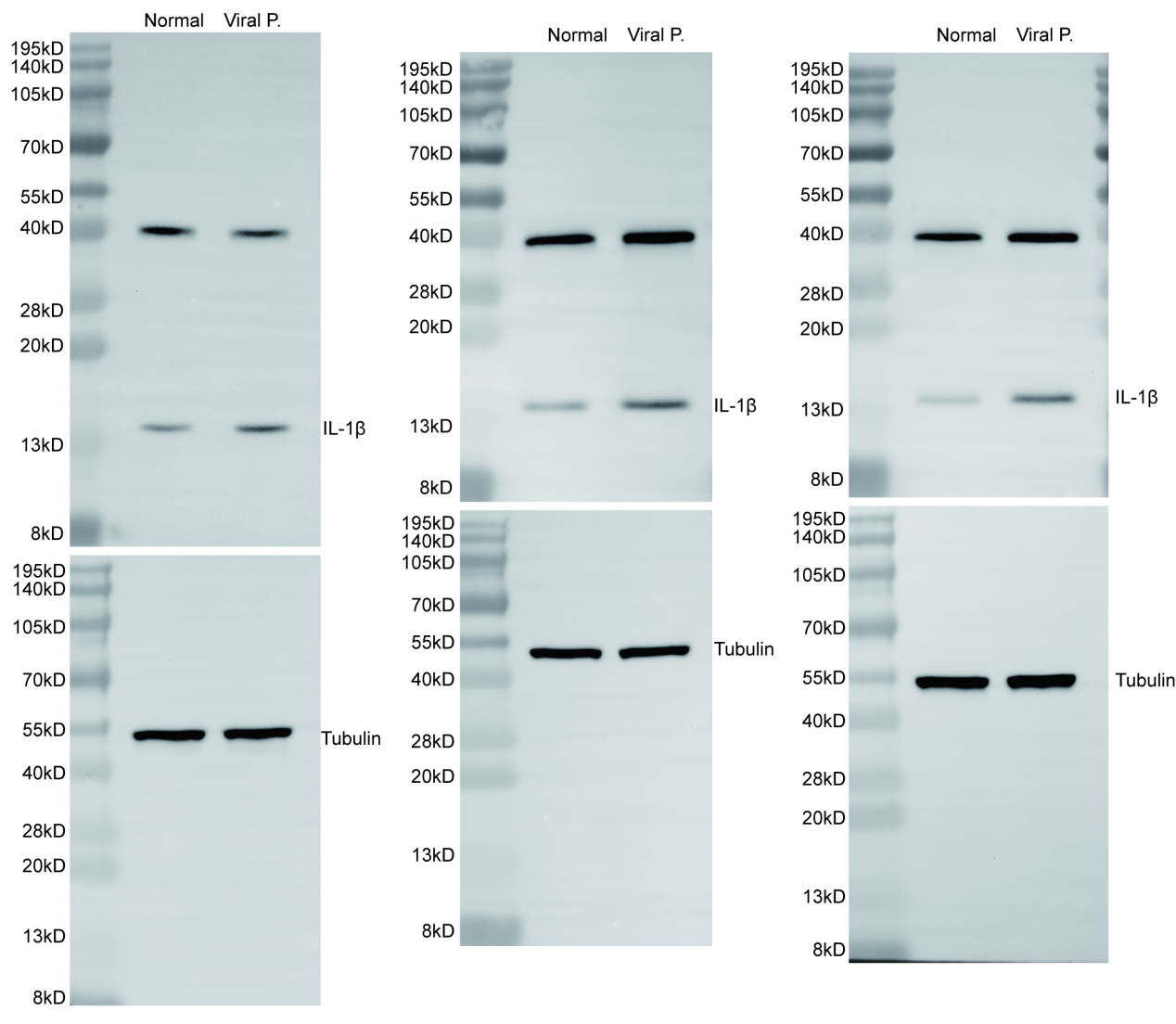
**
